# Bottom-up construction and screening of algae-bacteria consortia for pollutant biodegradation

**DOI:** 10.3389/fmicb.2024.1349016

**Published:** 2024-02-08

**Authors:** Zongting Cai, Esther Karunakaran, Jagroop Pandhal

**Affiliations:** ^1^Department of Chemical and Biological Engineering, The University of Sheffield, Sheffield, United Kingdom; ^2^Grantham Centre for Sustainable Futures, The University of Sheffield, Sheffield, United Kingdom

**Keywords:** algae-bacteria consortia, microbial system engineering, bioremediation, volatile organic compound, screening method

## Abstract

Microbial communities have been used as important biological tools for a variety of purposes associated with agriculture, the food industry and human health. Artificial engineering of microbial communities is an emerging field of research motivated by finding stable and efficient microbial systems. However, the successful design of microbial communities with desirable functions not only requires profound understanding of microbial activities, but also needs efficient approaches to piece together the known microbial traits to give rise to more complex systems. This study demonstrates the bottom-up integration of environmentally isolated phototrophic microalgae and chemotrophic bacteria as artificial consortia to bio-degrade selected volatile organic compounds (VOCs). A high throughput screening method based on 96-well plate format was developed for discovering consortia with bioremediation potential. Screened exemplar consortia were verified for VOCs degradation performance, among these, certain robust consortia were estimated to have achieved efficiencies of 95.72% and 92.70% and near 100% removal (7 days) of benzene, toluene, and phenol, respectively, with initial concentrations of 100 mg/L. VOCs degradation by consortia was mainly attributed to certain bacteria including *Rhodococcus erythropolis*, and *Cupriavidus metallidurans*, and directly contributed to the growth of microalgae *Coelastrella terrestris* (*R* = 0.82, *p* < 0.001). This work revealed the potential of converting VOCs waste into algal biomass by algae-bacteria consortia constructed through a bottom-up approach. The screening method enables rapid shortlisting of consortia combinatorial scenarios without prior knowledge about the individual strains or the need for interpreting complex microbial interactions.

## 1 Introduction

The majority of biological treatment technologies for pollutant remediation utilise bacteria due to their diversity and metabolic versatility in utilising a variety of substrates (Antizar-Ladislao, 2010; Numberger et al., 2019). Many well-characterised bacterial genera such as *Pseudomonas*, *Rhodococcus* and *Bacillus*, have been used to degrade a variety of toxic compounds including formaldehyde (Roca et al., 2008), benzene, phenol, and toluene (Reardon et al., 2000; Abuhamed et al., 2004) from wastewater, gaseous waste streams and polluted soils (Antizar-Ladislao, 2010; Lu et al., 2012; Numberger et al., 2019). Whilst bacteria dominate pollutant bioremediation, the inclusion of microalgae in the process presents attractive benefits. Microalgae are versatile organisms that can capture CO_2_ from biodegradation processes, providing oxygen in return to support bacterial enzymatic activites. Microalgal biomass also serves as a rich source of lipids for biofuel production, carbohydrates for ethanol and biogas (Chandrasekhar et al., 2023), and fibrous polymers for biodegradable plastics (Zanchetta et al., 2021). They can also provide high-value products like pigments (Stachowiak and Szulc, 2021) and vitamins (Arora and Philippidis, 2023). Given these benefits, microalgae are viewed as solar-driven cell factories for sustainable biomanufacturing.

Algae-bacteria communities are some of the most common and fundamental forms of mixed microbial systems in nature, in which, nutrient exchange is the most basic interaction associated with their co-existence. In aquatic environments, for example, algae are responsible for providing a large amount of dissolved organic carbon (DOC) that can work as the major carbon source for the survival of heterotrophic bacteria (Weinberger et al., 1994; Takemura et al., 2014). An early study by Humenik and Hanna (1971) showed that combined green alga *Chlorella* and bacteria in activated sludge resulted in sufficient algal-originated O_2_ supply for bacteria and increased algal protein production within a wastewater treatment (WWT) system. This early finding became the starting point of many studies motivated by seeking other superior properties over bacteria-only systems, such as self-supporting systems and enhanced nutrient removal (Min et al., 2011; Zhang et al., 2012; Inoue and Uchida, 2013). These studies followed a top-down approach for consortia design, i.e., using naturally occurring algae–bacteria consortia as the inoculant, which usually involves highly complex microbial communities.

Integrating individual strains to form multi-strain systems provides a bottom-up route of algae-bacteria consortia engineering. Usually, prior understanding and characterisation of the biological phenotype of the strains is vital. An early study by Mouget et al. (1995) suggested that two *Pseudomonas* strains isolated from laboratory algal cultures, *Pseudomonas diminuta* and *Pseudomonas vesicularis*, could stimulate the growth of co-cultured green microalgae *Scenedesmus bicellularis* and *Chlorella* sp., simply by providing CO_2_ and consuming oxygen to maintain a preferable condition for photosynthesis. As the knowledge base surrounding the algae-associated microbiome (or phycosphere) becomes more available, intentionally selected bacterial partners for specific algae species have emerged as a viable option for the bottom-up construction of algal-bacterial consortia. In another study, Dong and Zhao (2004) obtained 12.92 mg/L of astaxanthin production in a mixed culture of *Haematococcus pluvialis* and *Phaffia rhodozyma*, which was 3.5- and 11-fold higher than those in axenic cultures of the two strains, respectively. Wirth et al. (2015) mixed microalgae, *Chlamydomonas* sp. and *Scenedesmus* sp., with bacteria *Rhizobium* sp., and increased algal biomass yields by up to 24% as the result of O_2_-CO_2_ exchange. Due to the same mechanism, Papone et al. (2012) obtained enhanced lipid production in *Chlorella* sp. *KKUS2* by choosing fungi *Toluraspore YU5/2*, and *Toluraspore Y30* for co-culturing. Apart from wild-type strains, engineered bacterial strains have even greater potential for applications in algae-bacteria co-culture systems. For example, Therien et al. (2014) designed an artificial cross-feeding system consisting of glycogen synthase knockout mutant *Synechococcus* sp. 7002 that provides acetate to support lipid-producing *Chlamydomonas reinhardtii*. In addition to function enhancement and productivity promotion (Xie et al., 2013; Amin et al., 2015; Segev et al., 2016), the adverse interaction between certain algae and bacteria can also be utilised to address certain environmental challenges. For example, harmful algal bloom control can be achieved by bacteria that release algicidal molecules including 1-acetyl-β-carboline (Kim et al., 2015), Orfamide A (Aiyar et al., 2017) and urocanic acid (Kim et al., 2018; Zhuang et al., 2018).

Despite these achievement, challenges remain for both top-down and bottom-up approaches to form algae-bacteria consortia. Top-down approaches struggle with the specificity of target interactions and often fail to elucidate community interactions or underlying mechanisms, hindered by the sheer number of possible interactions. Efforts to bridge this gap using omics and evolutionary tools have been made (Okurowska et al., 2021; Qu et al., 2021; Si et al., 2022), but more efficient methods integrating top-down data with functional insights are needed for designing specific algae-bacteria consortia. In bottom-up approaches, predictability is the main hurdle. Microbiome biology is influenced by various ecological principles like competition (Griffin et al., 2004; Hibbing et al., 2009), ecosystem succession (Fierer et al., 2010; Jiménez et al., 2017), and mass cycling (Gralka et al., 2020), which shape microbial community assembly. While computational tools have advanced our understanding and prediction capabilities (Coyte et al., 2015; Zomorrodi and Segrè, 2016; Marsland et al., 2019; Daly et al., 2022), experimental validation remains crucial. The complexity of multi-species microbial systems, with diverse interacting components and pathways, leads to unpredictable system-scale behaviours (Gilbert and Henry, 2015). Olson et al. (2012) highlight the difficulty in designing or optimising complex microbial systems without extensive data. Consequently, most synthetic algae-bacteria communities studied are limited to two-member systems (Mujtaba et al., 2017; Contreras-Angulo et al., 2019), with multi-strain studies being rare (Le Chevanton et al., 2013).

Here we aimed to design a screening method to enable the identification of high-performance algal-bacterial consortia that are capable of biodegradation. We targeted some of the most recognised health-threatening volatile organic compounds (VOCs) related to biomass combustion (Kim and Shim, 2010; Wielgosiski, 2012) and the isolation of co-existing strains in a relevant polluted environment. This work is motivated by limited knowledge of how to construct stable synthetic algae-bacteria consortia and the absence of efficient methodologies accessible in most research laboratories. We followed a bottom-up route to integrate environmentally isolated microalgae and chemotrophic bacteria to form VOCs-degrading consortia. By implementing a microplate-scale screening method and measuring chlorophyll abundance in this closed system, a high throughput data generation approach was developed which facilitated rapid shortlisting of algae-bacteria combinations. In addition to algae growth data, our experimental design takes advantage of the consortia combination structure to give rise to two scoring indexes which uncovered the algae growth-promoting potential and functional stability of consortia without the need for interpreting complex interaction networks. The screening method exhibits fair predictive accuracy for consortia performance against technical error and variation of other potential affecting factors during up-scaling, evidenced by the quantification of algae growth and VOCs degradation in flask-grown exemplar consortia. In addition, an end-point algae-bacteria community structure analysis was conducted to provide insight into the association between bacterial strains and the observed behaviour in exemplar consortia.

## 2 Materials and methods

### 2.1 Synthetic VOCs feeds

The selection of VOCs was based on the characterisation of the air pollutants emitted through the industrial biomass combustion field. Benzene, toluene, and phenol were selected as representative aromatic hydrocarbons and tetrahydrofuran was selected as representative furanic compounds for their abundance in waste streams (Kim and Shim, 2010; Wielgosiski, 2012). All four selected chemicals have limited solubility in water (Supplementary Table S1) at room temperature, which allows the preparation of 2,000 mg/L (500 mg/L each compound) strength VOCs aqueous solution stock by directly dissolving the four chemicals in HPLC grade sterile deionised water (Thermo Fisher).

### 2.2 Acquisition of bacteria and microalgae strains

A site under prolonged exposure to exhaust and crude oil products was chosen for the enhanced possibility of isolation and enrichment of bacteria and microalgal strains that are either tolerant or even able to degrade the VOCs of interest. Soil samples were taken from road verges with apparent oil spillage at 53 °23 ′11.88′′ N, 1°28 ′46.02′′ W. Around 50 grams of soil samples were taken from the chosen site at a depth of 3–5 cm below the surface which was preserved in laboratory fridge at 4°C before processing.

Approximately 10 g of soil sample aliquot were suspended in sterile PBS solution with a final volume of 30 mL, which was then split into 6 × 5 mL and transferred to triplicated heterotrophic and autotrophic flasks with equal culturing volumes of 50 mL following the culturing scheme listed in Table 1.

**Table 1:** Media and VOCs concentration of different culture flasks.

Item	Media	Light scheme	VOCs concentration (mg/L)			
			B	T	P	THF
Heterotrophic flasks	PBS media + LB broth	Dark (24 h)	10	10	10	10
Autotrophic flasks	3 N-BBM + V	Light (24 h)	10	10	10	10

LB broth final concentration 2 g/L in the media; 3 N-BBM + V recipe is available at Media Recipes-CCAP (Bischoff, 1963); B, Benzene; T, Toluene; P, Phenol; THF, Tetrahydrofuran.

All the flasks were sealed with breathable films and contained in a 30 × 30 × 15 cm plastic box with a porous lid that allows aerobic conditions. The selective culturing was performed at room temperature of 25°C for 4 weeks. Growth status was visually checked daily. To compensate for the VOC evaporation and nutrient consumption, partial media refreshments (5 mL) were performed every 2 days but to avoid diluting the biomass in the culture, this was only preferably performed with flasks that showed obvious signs of microorganism growth including turbidity and colours changes. After 4 weeks, another four-week sub-culturing was performed following the same incubation and maintenance scheme. At the end of the 8 weeks, 5 mL samples were taken from each flask and centrifuged (2000 *g*, 5 min). Acquired biomass pellets were washed with PBS buffer followed by 10-fold dilutions and plating on the same media in agar which were then incubated for 1 week under room temperature with or without illumination (24 h, 7 days) depending on the initial heterotrophic/autotrophic schemes of inoculants. Any emerging bacteria and microalgae colonies were presumptively classified according to their morphologies and cell characteristics (detailed in Supplementary Figures S1–S3).

Classified bacteria and microalgae colonies were collected and purified following the limiting dilution method described by Lo Surdo and Bauer (2012) and subcultures were further purified using the streak plate method. To obtain axenic microalgae culture, 20 μL volumes of diluted algae subcultures were spread evenly on Petri dishes containing LB agar, and incubated for 72 h under dark conditions, room temperature. This process was continuously repeated until the cultures were confirmed to be bacteria-free, namely, having no sign of any microbial growth on the plate. For bacteria isolates, any subcultures showing identical colony morphology were considered axenic.

Apart from environmental isolates, *Pseudomonas putida KT2440*, a well-known VOCs degrading strain (Jiménez et al., 2002) was acquired from the Department of Animal and Plant Science, University of Sheffield.

### 2.3 Strain characterisation and identification

Axenic algae isolates were further characterised for their growth rate and VOCs tolerance limits using a gradient of VOCs concentration from 10 mg/L to 100 mg/L of each compound. All bacterial isolates, prepared in duplicates, were inoculated for 24 h in LB media followed by a revival test on Petri dishes containing LB agar to verify their VOC-resistant traits. These isolates were also cultured in PBS solution containing 100 mg/L of each VOC as the sole carbon source, where any observed growth (Supplementary Figure S4) serves as an preliminary indication of potential VOC degradation. The choice of VOCs concentration was guided by prevalent BTEX degradation studies (Kim et al., 2008; Jiang et al., 2015). Any bacteria isolate that fails to revive would not be considered. Parallel to the characterisation, all pre-screened bacteria isolated were further identified by 16 s rRNA gene sequencing (for bacteria),and 18 s, ITS1-5.8S-ITS2 regions of rRNA gene sequencing (for algae). Bacteria templates DNA were obtained via the heating-lysis method (Englen and Kelley, 2000) under 98°C for 5 min in 20 μL of nuclease-free water (QIAGEN) using a thermocycler (Applied Biosystems^™^). Microalgae template DNA was extracted from pellets of liquid culture (5 days culture) through 10 min incubating under room temperature in 20 μL of dilution buffer aliquots provided in Phire Plant Direct PCR Kit (Thermo Scientific^™^). To ensure successful DNA amplification, two different sets of primer pairs were selected for the bacteria sample and four pairs of primer were selected for the microalgae sample. PCR of bacterial and algal template DNA was performed using PCRBIO^®^ Taq Mix Red kit and Phire Plant Direct PCR Kit (Thermo Scientific^™^), respectively, following the kit instructions. All DNA extractions and PCRs were performed in duplicate. Details of the PCR program setting, and primer sequence are listed in Supplementary Table S2.

The PCR products were checked by gel electrophoresis on 1% ultrapure agarose gel (Invitrogen^™^) prepared using Tris-acetate-EDTA (TAE) buffer stained by ethidium bromide (1%). Amplification products that match specific lengths were collected and further purified using the QIAquick Gel Extraction Kit (QIAGEN) following the protocol given in the manual. The concentrations of extracted DNA samples were measured using a microvolume spectrophotometer (NanoDrop^™^, Thermo Scientific). Bacterial DNA samples were submitted to the Sheffield Microarray/Genomics Core Facility (The University of Sheffield, UK) and sequenced using a 3,730 DNA analyser (Applied Biosystems^®^). Microalgal DNA samples were sequenced by Azenta Life Sciences (formerly GENEWIZ^®^). All sequencing was conducted bidirectionally and was searched for their species or genus identities using NCBI-BLAST.

### 2.4 Algae-bacteria consortia screening

To test as many consortia as possible while avoiding generating a large amount of combinations scenarios, bacteria strains were first shortlisted according to (1) their ability to degrade VOCs as the carbon source for growth; and (2) whether repeated strains are included, i.e., strains of the same genera and having similar colonies morphology. The shortlisted microbes were grouped to form consortia, each of which consisted of one algae strain, one VOC-degrading bacteria strain (degrader), and at least one bacterium without any obvious VOC degradation traits (non-degrader).

Microalgae inoculant was distributed into 96-well plates with even cell densities controlled at approximately 1,000 cells per well except for blanks. Microplate wells that were only inoculated with algae were marked as controls. Bacteria overnight cultures in LB broth were pipetted (1 μL per strain) separately into the corresponding wells according to the predetermined combination scenarios (Supplementary Table S5), which ensured that each combination is prepared in at least duplicate and that the remaining wells are appropriately allocated for controls, i.e., blanks and consortia containing only algae and degraders. After inoculation, each well was topped up to a 200 μL final culture volume using 3 N-BBM medium containing benzene, toluene, phenol and tetrahydrofuran, 100 mg/L per compound. Inoculated 96-well plates were covered with optical sealing films (SealPlate Sealing Film, sterile, Elkay) to securely seal individual wells, while leaving approximately 200 μL headspace. All plates were kept at 25°C with even and continuous luminance for 7 days. Although cultivation parameters such as growth media, temperature, pH, vessels, and starter ratios can impact microbial interactions, these were not varied in this screen.

Bulk chlorophyll concentrations were chosen as the only indicator for algae growth of the consortia. Chlorophyll concentration was measured in terms of fluorescence (em:690 nm, ex:390 nm) using Spark^®^ 10 M multimode microplate reader (Tecan). To check the correlation of algae cell density with the chlorophyll fluorescence signals, a trial measurement was performed using an axenic algae culture of the candidate algae strain, an algae-bacteria mix culture and an axenic culture of a non-candidate algae strain (*Deuterostichococcus epilithicus*) as external control. All three samples were pre-grown for 7 days and diluted two fold to form a gradient of algae cell densities in the 96-well plate, which were later fitted with corresponding fluorescence data for measurement validation (Supplementary Figure S8).

### 2.5 Screening data processing

Fluorescence data from the screening experiment were normalised by subtracting the average data of blanks and were then standardised by rescaling to a (0, 1) range using Equation 1.


(1) $$X_{\mathrm{rescale}}=a+\left[\frac{X-X_{\text{min}}}{X_{\text{max}}-X_{\text{min}}}\right]\left(b-a\right)$$

*X* refers to the values that are rescaled to an arbitrary interval [*a b*].

The individual consortium was assessed for its performance according to the value of its growth score (***GS***), defined as the difference in its standardised fluorescence data versus the average fluorescence data of controls, using Equation 2.


(2) $$GS=\overset{n=7}{\underset{0}{\sum }}\left(X_{\mathrm{Day}\;n}-C_{\mathrm{Day}\;n}\right)$$

*GS*: Difference in fluorescence data between of a consortium ( X ) versus that of the control group ( C ) at a specific sampling time (*Day 0* to *Day 7*).

**Figure 1:**
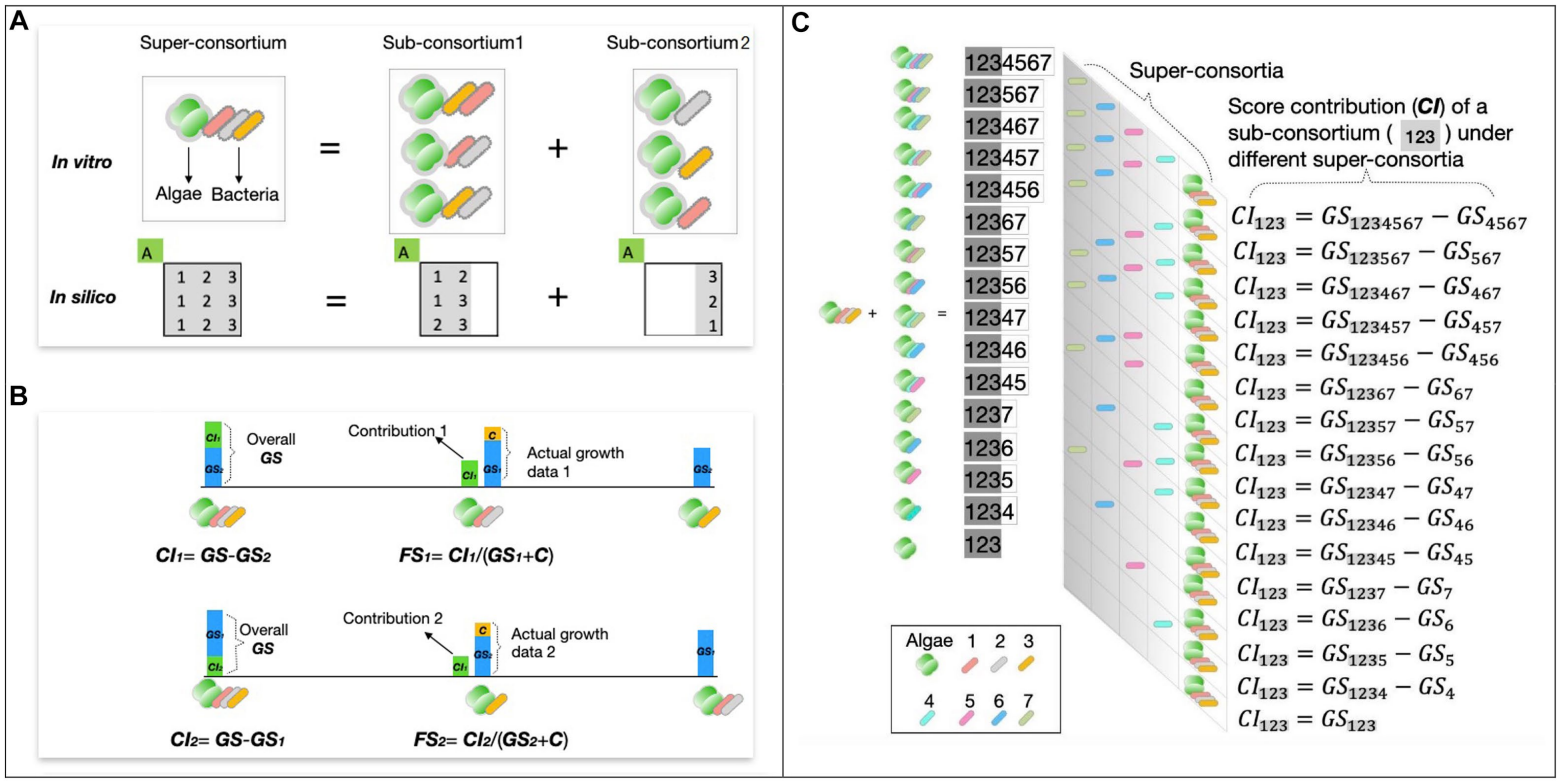
Screen data processing and concept demonstration. **(A)** Example of a 3-bacteria consortium and its sub-consortia. The consortium can be seen as a super-consortium and split into 3 different pairs of sub-consortia, with corresponding *in silico* expression as matrix manipulation. Each row of the matrix represents an option for breaking down the original consortium demonstrated in the *in vitro* diagram. Letter ‘**A**’ refers to algae and numbers 1, 2, and 3 represent co-cultured bacteria strains. **(B)** Conceptual definitions for screen parameters ***CI*** and ***FS***. **(C)** Hierarchical structures of multiple ***CI*** of the same consortium under different super-consortia.

Besides, any consortium with bacterial strain numbers >2 can be seen as a **super-consortium** consisting of **sub-consortia** that have different contributions of ***GS*** to its **super-consortia** as demonstrated in the example (Figure 1A). This gives rise to two additional parameters, growth contribution index (***CI***) and functional stability (***FS***), which reflect the strength and stability of the algae growth-promoting effect of different bacteria combinations (Figure 1B).

The ***CI*** is defined as the difference between the ***GS*** of the **super consortium** and the ***GS*** of the counterpart or pairing **sub-consortium** Equation 3. Likewise, the ***FS*** of a given **sub-consortium** with a specific **super-consortium** is defined as the ratio between its ***CI*** to the super-consortium and its own ***GS*** (Equation 3).


(3) $$\left\{ \begin{array}{c} \mathrm{Cons}=Sub1\cup Sub2\\ CS_{1}=GS-GS_{2}\\ CS_{2}=GS-GS_{1}\\ FS_{1}=\frac{CI_{1}+C}{GS_{1}+C}\\ FS_{2}=\frac{CI_{2}+C}{GS_{2}+C} \end{array}\right.$$

Cons,Sub1andSub2 are the superset and its two subsets representing the respectively, which represent the sup. $CI_{1},CI_{1}\;\mathrm{and}\;FS_{1},FS_{2}$ are the contribution and functional stability indexes of Sub1andSub2,repectively . $GS,GS_{2},GS_{1}$ refers to the actual growth scores of Cons,Sub1andSub2 . C is the sum of 7-day fluorescence data of axenic algae control.

The combinational feature of the consortia in this study allows a **single consortium** to have multiple choices of **super-consortia**, which mathematically exhibit hierarchical structures that include multiple ***CI*** (Figure 1C), as well as ***FS*** index under different super-consortia. To present the overall growth contribution and function stability of the given consortium, ***CI*** and ***FS*** calculated under multiple super-consortia, were processed using Equation 4.


(4) $$\left\{ \begin{array}{c} CI=\frac{1}{2^{7-s}}\times \overset{7-s}{\underset{n=0}{\sum }}\overset{\left(\begin{array}{c} 7-s\\ n \end{array}\right)}{\underset{i=1}{\sum }}CI_{i}\\ FS=\frac{1}{2^{7-s}}\times \overset{7-s}{\underset{n=0}{\sum }}\overset{\left(\begin{array}{c} 7-s\\ n \end{array}\right)}{\underset{i=1}{\sum }}FS_{i} \end{array}\right.$$

CI and FS are the overall growth contribution index and functional stability index of a given consortium, i.e., average value of CIi and FSi which are the single contribution and functional stability indexes obtained under different super-consortia; Consortium size s refers to number of non-degrader bacteria strains in this given consortium.

### 2.6 Combination significance and single strain effect test

To analyse how bacteria combinations affect consortia behaviours, student t-test was performed against ***GS***, ***CI***, and ***FS*** values. For any combination of certain bacteria, the screened consortia automatically fall into two collections, (1) that either contain the same bacteria member or (2) without these bacteria. The two collections served as two sample sets for double-tailed t-tests (non-paired) with a value of p threshold set as 0.05 for determining significant (*p* ≤ 0.05) and non-significant (*p* > 0.05) combinations which were further adjusted via Benjamini-Hochberg correction (Benjamini and Hochberg, 1995) to minimise the false positive rate (FPR). The effects of single bacteria strains were analysed by comparisons on consortia with and without each strain. ***GS***, ***CI*** and ***FS*** values subject to the presence and absence of each strain were normalised by rescaling within the range of −1 to 1 using the same method specified in Equation 1. In addition, the statistic correlations among the three parameters, together with their correlation between growth data of flask cultures, were evaluated via Pearson correlation coefficients (PCC) with *p* = 0.05 as the significant threshold.

### 2.7 VOCs biodegradation analysis

Using ***CI*** as the main criterium, consortia with high, medium and low ***CI*** values were chosen as exemplars. Prepared in duplicates, these exemplar consortia were cultured for 7 days in 60 mL of medium with the same VOC concentration as adopted screening experiments, using 100 mL gas-tight flasks assembled with PTFE septum aluminium crimp seals. The incubation conditions of 25°C, continuous illumination, and orbital shaking (120 rpm/min) were used. 3 mL of liquid culture samples were taken from each flask daily using sterile syringes and needles after vortexing. To separate residual VOCs from the aqueous phase, 1 mL aliquots were passed through 0.22 μm glass fibre syringe filters and transferred into glass vials, followed by liquid–liquid extraction using 0.5 mL of HPLC grade dichloromethane (Thermo Fisher Scientific) through adequate mixing on a tube rotator for 12 h. The organic phase containing analytes VOCs was transferred into 2 mL autosampler vials which were processed by Gas Chromatography and Flame ionisation detector (GC-FID) platform (Thermo Fisher Scientific) equipped with a HP-5 column (Inner Diameter:0.32 mm, Film thickness:0.25 μm, length 30 m, Agilent). The chromatography programme was set as follows: initial oven temperature 35°C for 15 s, then rise to 185°C at a ramp of 80°C per minute. The injector and detector temperature was set as 250°C and 300°C, respectively. Samples were injected as 1 μL aliquots in split mode with a slit ratio of 8.0:1. To enhance separation resolution, high-purity helium was used as carrier gas with a flow rate of 1.0 mL/min in the first 45 s after sample injection, then increased to 2 mL/min with a ramp of 5 mL/min.

Notably, the GC data may not fully represent the actual VOC quantities in the incubation flask due to VOC redistribution between liquid samples and headspace air, and sampling loss. Therefore, the direct GC data were corrected using Henry’s Law, under the assumption that all four VOC compounds maintain a constant extraction efficiency, or Partitioning Coefficient, in water/DCM systems under equilibrium conditions (Moldoveanu and David, 2015a,b; Amézqueta et al., 2020). Detailed calculations of this correction are presented in Session 6, Supplementary material 1.

### 2.8 Microbial community structure analysis

Algae populations were quantified via optical density set as absorbance at 685 nm (OD_685_) using a spectrophotometer. To enhance comparability with screening results, daily OD_685_ data of flask-grown exemplar consortia were normalised using the control data and then summed to reflect the accumulative feature of the total algae biomass in 7 days. The bacterial population of individual strains in each exemplar consortium, including those in biological replicates, were quantified via quantitative polymerase chain reaction (qPCR) using the remaining 2 mL sample aliquots. Consortia biomass pellets were obtained by high-speed centrifugation (10,000 *g*, 1 min) and were washed using nuclease-free water to remove salts and residual phenolic compounds. Full consortia DNA extraction was performed using PCRBIO Rapid Extract Lysis Kit (PCR Biosystems) following the kit instructions. To ensure thorough DNA extraction, an excessive amount of reagents were used, combined with extended incubation time (30 min) for both the cell lysis (75°C) and proteinase deactivation (95°C) and additional vertex steps in between. Obtained consortia genome DNA extracts were directly used for qPCR. For calibration standards preparation, 10^−1^ to 10^−5^ dilutions were performed for individual strain genome DNA extracted from 100 mL of axenic cultures for algae (7 days culture) and 100 mL of each bacteria (overnight culture) following the same DNA extraction process and additional cleaning step using ethanol acetate precipitation method specified by Sambrook et al. (1989). The initial numbers of DNA molecules in qPCR calibration standards were determined using Equation 5 (Karunakaran et al., 2016) based on known DNA genome concentrations obtained using a microvolume spectrophotometer (NanoDrop^™^, Thermo Scientific).

Bacteria cell densities in the consortia were estimated as DNA molecule numbers per qPCR sample after correction against 16S rRNA gene copy number variations per species using Ribosomal RNA operon copy numbers data obtained from the rrnDB database (Stoddard et al., 2015).


(5) $$N=\frac{6.022\times 10^{23}\times C}{660\times L}$$

N : Number of DNA molecules per μL of sample; C : DNA concentration (g/μL); L : Length of the known strain genome (bp), data obtained from GenBank (genome statistics),* For strains without species-level identity, median total genome length of the related genus was taken.

All qPCR reactions were set up in triplictes for each DNA extracts, and were conducted in 96-well PCR plates (Sarstedt) using qPCRBIO SyGreen Mix Lo-ROX kit (PCR Biosystems) on a 7,500 Fast Real-Time PCR System (Applied Biosystems^®^, Thermo Scientific) with strain-specific primers pairs which target bacterial 16S rDNA. For the four *Rhodococcus* strains (non-degrader *7*, degrader *B*, *E* and *F*), primers were designed based on the catechol 1,2-dioxygenase gene (CatA) gene, considering the prevalent application of assay based on catA gene in differentiating species within this genus (Táncsics et al., 2008; Iminova et al., 2022) and the possible inefficiency of 16S rDNA for identifying these strain (Ladrón et al., 2022). The sequences of primers are detailed in Supplementary Table S7. To increase the amplification specificity, the PCR program for all primer pairs was set to be 80 cycles with an initial denaturing at 95°C for 30 s, and 5 s of denaturing at 95°C followed by Primer annealing and extension at 72°C for 30 s per cycle, unless specified otherwise. The reaction volumes were all set as 10 μL.

## 3 Results and discussion

### 3.1 Microbial enrichments and identification

Environmental isolation, together with one *Pseudomonas putida KT2440* obtained from lab collection, resulted in a total of 26 bacteria (Supplementary Figure S1 and Supplementary Table S1) with VOC-resistant features and 6 exhibited potential VOC-degrading phenotypes. Two soil algae isolates obtained from autotrophic cultures, were characterised for their growth characteristics (Supplementary Figures S5, S6). One strain which survived up to 200 mg/L of each VOC was selected as the candidate algae strain (Supplementary Figure S7).

The 16S rDNA sequencing results revealed the dominance of gram-negative (GN) *Pseudomonas* and gram-positive (GP) *Rhodococcus* in these 26 bacteria isolates (Supplementary Figure S1), both known for their capability to degrade aromatic compounds and use them as growth substrates (Kauppi et al., 1998; Cébron et al., 2008). Additionally, two isolates of *Delftia*, a GN bacterium capable of degrading low molecular weight phenolic compounds and aniline (Sheludchenko et al., 2005; Juárez-Jiménez et al., 2010), were also classified as degraders. These genera of bacteria possess multiple enzyme systems for breaking down aromatic compounds (Kauppi et al., 1998; Shigematsu et al., 2003; Patrauchan et al., 2008) with *Rhodococcus jostii RHA1* predicted to have over 200 oxygenases and 30 pathways for aromatic compound catabolism (McLeod et al., 2006).

Although bacterial isolates like *Plantibacter*, *Achromobacter*, and *Ochrobactrum anthropic* did not exhibit direct signs of VOC degradation, they demonstrated VOC resistance in this experiment, along with other beneficial characteristics such as plant growth promotion identified in previous research (Chakraborty et al., 2009; Meng et al., 2014; Mayer et al., 2019; Jiménez-Vázquez et al., 2020), underscore their potential significance in an algae bacteria consortium. The employment of these strains may result in the broader unknown role beyond VOC degradation.

However, considering the theoretical possibility of 2^26^ (or 268,435,455) combinations, it was impractical to explore all of these *in-vitro*. To manage this complexity, a final collection of 6 VOC-degrading bacteria (degraders) and 7 non-VOC-degrading bacteria (non-degrader) and *Coelastrella terrestris* (Table 2) were chosen as the “building blocks” for the algae-bacteria consortia which resulted in 6 × 2^7^ = 768 different combinations. Growth data of a total of 1920 samples were measured, including biological duplicates for each consortium (768 × = 1,536), 90 controls, 102 blanks and 32 × 6 consortia containing only algae and degraders. Details of microplate layouts are presented in the Supplementary Table S5.

**Table 2:** Strains involved in constructing algae-bacteria consortia.

Microbe type	Strain label	Identification	Sequence accession number(s)
Bacteria: VOC degraders	A	*Pseudomonas fluorescens*	PP106129
	B	*Rhodococcus erythropolis*	PP106147
	C	*Pseudomonas* sp.	PP106139
	D	*Delftia* sp.	PP106142
	E	*Rhodococcus sp_1_.*	PP106148
	F	*Rhodococcus sp_2_.*	PP106152
Bacteria: VOC non-degraders	1	*Pseudomonas syringae*	PP106131
	2	*Agromyces atrinae*	PP106151
	3	*Cupriavidus metallidurans*	PP106143
	4	*Ochrobactrum anthropi*	PP106150
	5	*Plantibacter flavus*	PP106144
	6	*Plantibacter* sp.	PP106153
	7	*Rhodococcus sp_3_.*	PP106149
Microalga	Ag	*Coelastrella terrestris*	PP106155

### 3.2 Monitoring algae growth as a proxy for consortia performance in the screen

**Figure 2:**
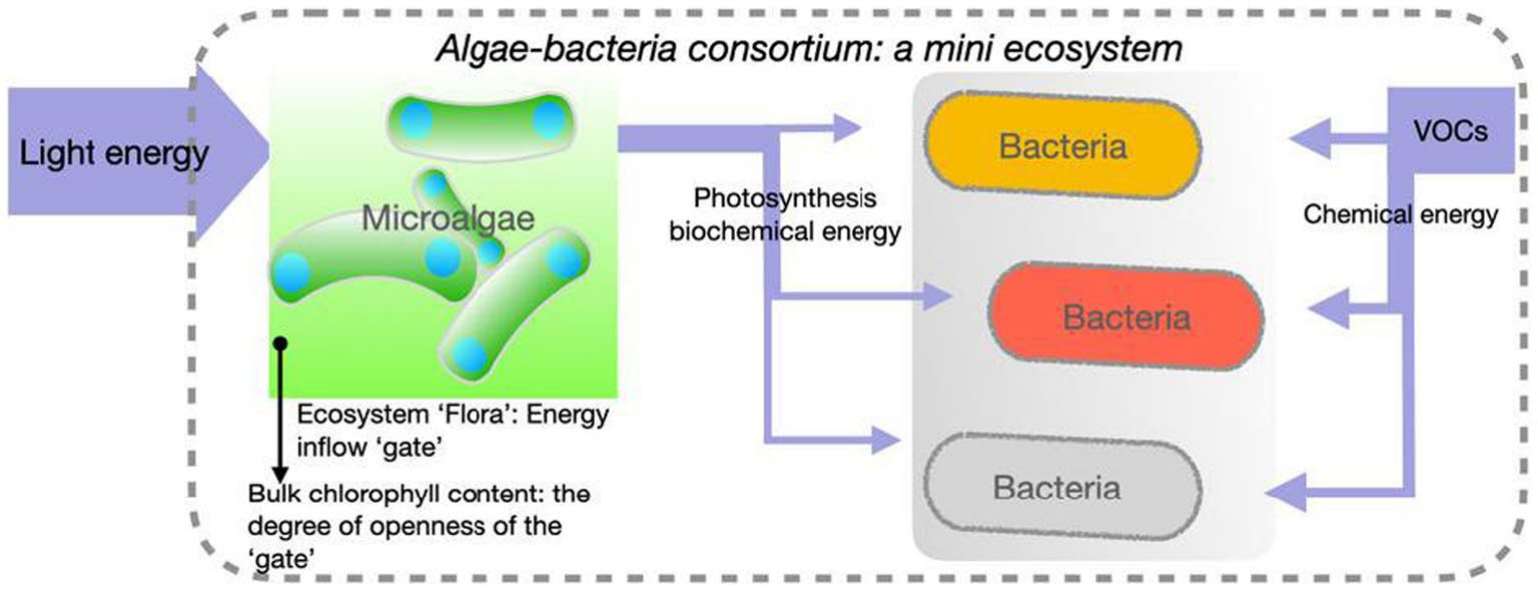
An ecosystem model of the algae-bacteria consortium in the high throughput screen. An algae-bacteria consortium and its containing environments is an ecosystem where the majority of carbon sources for the growth of the entire microbial community are from VOCs degradation by bacteria with algal photosynthesis as the main energy input and algae biomass as the final VOC-originated carbon sinks.

The screening experiment was performed as a closed system without air exchange, therefore the axenic algal control samples showed no growth due to their autotrophic nature and the lack of access to atmospheric CO_2_ as a carbon source. Bacterial degradation of VOCs, therefore, determines the availability of CO_2_ as a carbon source, which directly limits the maximum potential growth of algae. The VOCs per litre of medium contributed only a theoretically 16.12 kJ of chemical energy under aerobic conditions (NIST Chemistry WebBook, n.d.) and 27.16 mmol of CO_2_ equivalent, the fixation of which requires at least 127.11 kJ of photonic energy under the 10% of photosynthetic efficiency (PE), the theoretical upper limit of microalgae (Roux, 2016; Masojídek et al., 2021). For this reason, actual algae growth mainly reflects the amount of photonic energy fixed into consortia, which suggests the consortium’s capability for energy capture, primary production and suitability for scale-up. This mimicked closed ecosystem is hypothetically illustrated in the conceptual diagram (Figure 2).

### 3.3 Strains configuration for screening

By categorising characterised bacteria into VOC degraders and non-degraders, it is possible to significantly reduce the screening load by minimising the members of all possible combinations while analysing as many non-repeated consortia as possible. This helps to balance high throughput and analysis rate. Although VOC degradation may emerge among non-degrading bacteria because of microbial interactions, the ‘algae + degrader + non-degrader’ structure is expected to increase the possibility of discovering high-performance VOC removal consortia.

### 3.4 Comparison of screening results

**Figure 3:**
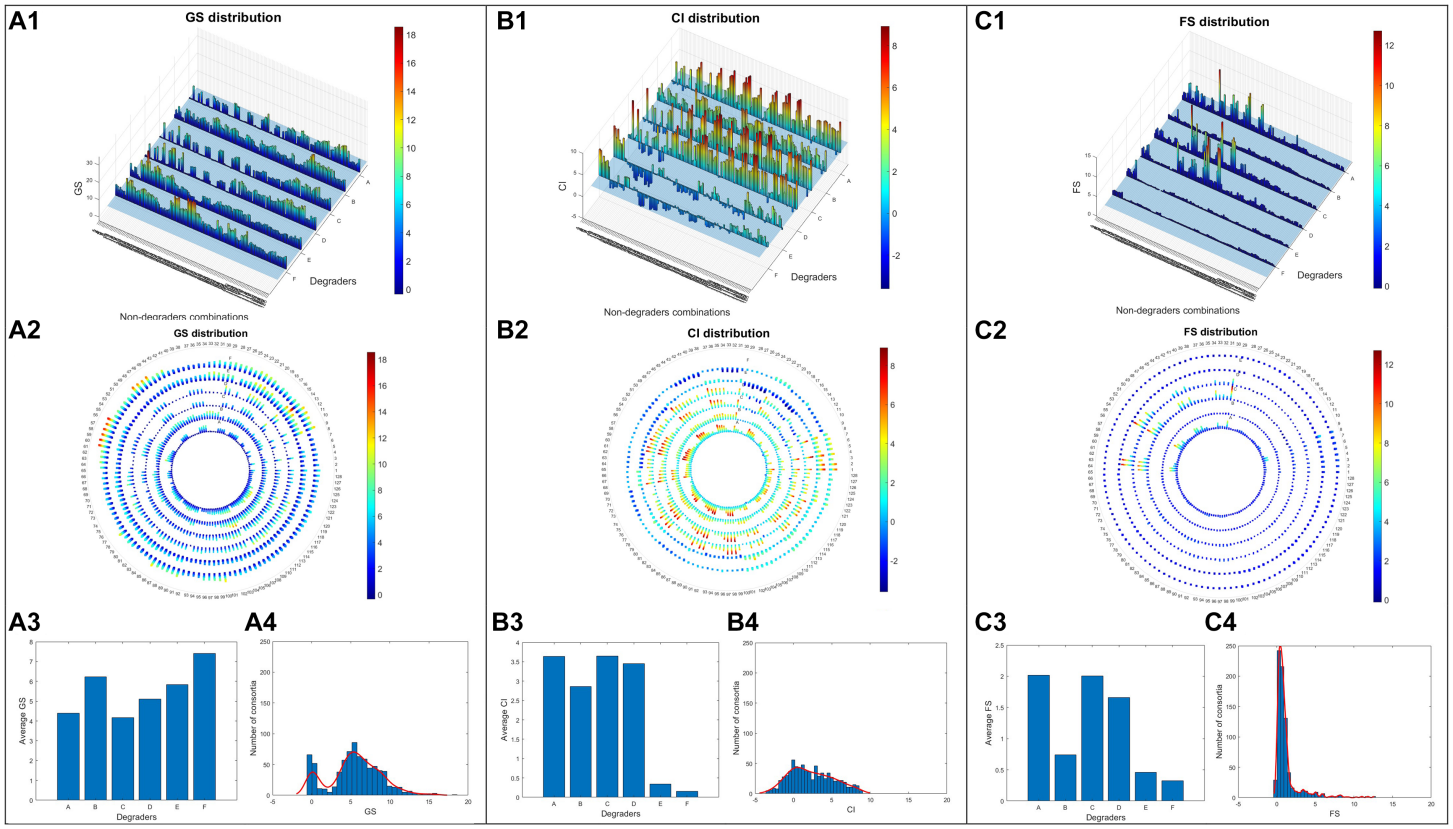
Screening data of 768 different consortia. Growth score data (***GS***) were visualised in the bar chart **(A1)**, polar plot **(A2)**, average values under 6 different degraders **(A3)** and demonstration of ***GS*** value distribution via histogram **(A4)**. The same visualisations are also adopted for parameters ***CI* (B1–B4)** and ***FS* (C1–C4)**.

Distributions of the three consortia assessment parameters across the 768 consortia are visualised in Figure 3. The ***GSs*** of 768 consortia exhibited a bimodal distribution, characterised by 679 positive values and 87 negative values (Figure 3A4). Since ***GS*** was based on a direct comparison of chlorophyll accumulation in tested consortia versus axenic algae control, its distribution showed that most consortia had more algae growth than the control. Consortia with high algae growths were found to be associated with two *Rhodococcus* strains (Degraders B and F), and contrastingly, two *Pseudomonas* strains, (Degraders A and C), were found in some of the consortia with the lowest growth. Non-degrader ***3*** (*C. metallidurans*) had a stronger association with high-***GS*** consortia by presenting in 62.8% (241/384) of those with ***GS*** value higher than 5.59 (median), while other non-degraders were present in 50% or less. ***GS***-negative consortia are distinctly characterised by the absence of degraders B (*R. erythropolis*), E (*Rhodococcus sp_1_*) and F (*Rhodococcus sp_2_*) and non-degrader ***3***.

The distribution of ***CI*** (Figure 3B4) spanned both negative and positive values, with a slight trend of being centred around zero. This suggests the existence of both algae growth promotion and inhibition across the screened consortia, a greater number (587) of which, however, had neutral effects on algae growth. Unlike ***GS***, the comparative nature of ***CI*** allows it to uncover the behaviour of a consortium against the potential influence of different co-cultured bacterial strains while also improving the measurement accuracy due to an increased number of observations, which led to very distinct patterns between ***GS*** and ***CI*** of the same consortia. Interestingly, positive ***CI*** values were found evenly in consortia containing degraders A, B, C and D, while high ***CI*** values (≥ 1.97, median) seem to be subject to the presence of non-degrader 3. In contrast, ***CI*** values were significantly lower in consortia containing degraders E and F among which half (138/256) were found negative. This could be explained by their high algae growth promotion effects being dominant and leaving little room for further effects beyond their original contribution. Interestingly, consortia based on another *Rhodococcus* (degrader B) were found to have much higher ***CI*** values despite already having high ***GS*** values. This suggests the potential existence of special positive interactions associated with this degrader being synergistically improved.

***FS*** uncovered extra information about the stability of a consortium by assessing the difference between its ***CI*** and ***GS***. Theoretically, stable consortia are relatively independent systems with unchanged behaviour when co-cultured with different bacteria and their ***GS*** and ***CI*** values tend to be close to each other, thus having ***FS*** values close to 1. In contrast, ***FS*** values deviating from 1 indicate consortia of weaker resilience which are more subject to the influence of additional bacteria and end up in either algae growth promotion (***FS*** > 1) or growth inhibition (***FS*** < 1) effects. Most of the screened consortia (517) had low stability with ***FS*** values lower than 1 featured by a peak (Figure 3C4) lying between the range of 0 and 0.6. The trailing pattern of ***FS*** (Figure 3C4) towards higher values greater than 1 indicates that fewer consortia (249) had synergistic algae growth promotion effects and were closely associated with degraders A, C and D (Figures 3C1,C2). ***FS*** value in consortia based on the three *Rhodococcus* degraders (B, E and F) were homogeneously below 1, indicating that they were not significantly affected by any synergistic effects regardless of the varying combinations of non-degraders. A few stable consortia were found to be strongly related to degrader B, and non-degrader 3 (*C. metallidurans*) which had average ***FS*** values close to 1.

### 3.5 Combination significance and single strain effect test

**Figure 4:**
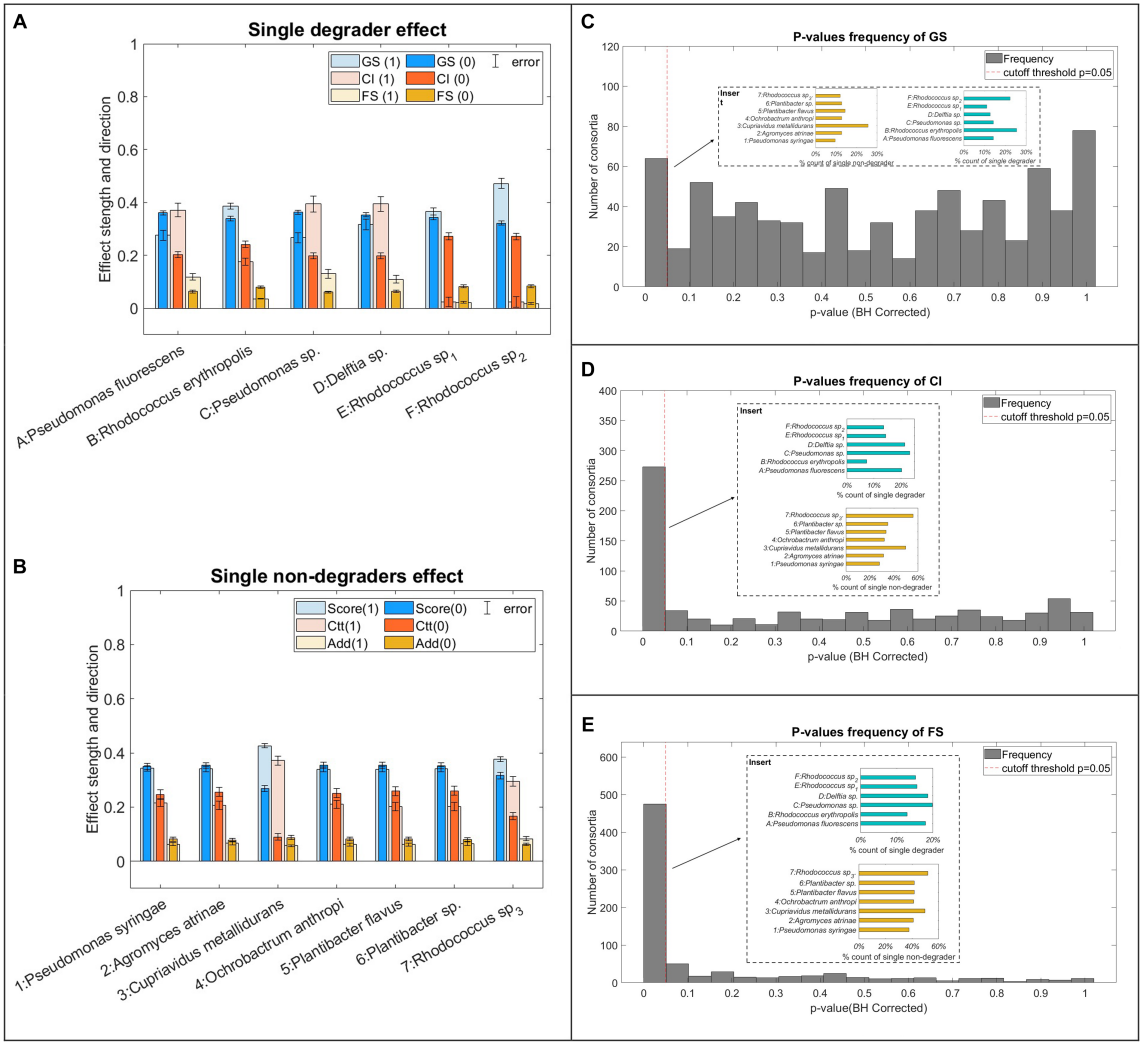
Combination significance test result. **(A,B)** Effects on the three parameters. The bar charts presented the normalised value of the three parameters with (light colour, marked as logic 1) and without (dark colour, marked as logic 0) specific strain. **(C–E)** Significance test on all possible combinations for ***GS***, ***CI***, and ***FS*** where histograms show the number of consortia hitting the *p*-values < 0.05 threshold (HB-corrected). Number of replicates?

Significance test results (Figure 4) revealed how the three parameters vary as the bacteria combinations change. After false positive rate (FPR) correction, ***GS*** was only found most affected (*p* < 0.05) by 63 consortia (Figure 4C) which are associated with the presence/absence of degrader B, F, and non-degrader 3 (Figure 4C, insert). ***CI*** values vary significantly under the presence and absence of 273 consortia wherein degraders A, C and D, and non-degraders 3 and 7 appear to be the primary bacteria strains that led to ***CI*** variation. A larger number (475) of consortia were found to significantly affect ***FS*** values. These consortia were featured by the absence of Degrader B. Non-degraders, 3 and 7.

The effect of individual strains (Figures 4A,B) indicates that two *Pseudomonas* (degrader A, C) had a very similar effect on all three assessment indexes. Specifically, consortia involving A and C tend to have lower ***GS*** but higher ***CI*** and ***FS*** compared to consortia that do not contain the two strains. This trend opposes what was observed in consortia containing *Rhodococcus* degrader (degrader B, E and F) which are featured by higher ***GS*** and significantly lower ***CI*** and ***FS***. *Delftia* (degrader D) had a similar effect on ***CI*** and ***FS*** to the two *Pseudomonas* degraders but higher ***GS***. Among non-degraders, *C. metallidurans* had the strongest effects on the consortia, evidenced by significantly higher ***GS*** and ***CI*** and reduced ***FS*** values associated with its presence. Also, non-degrader 7 resulted in higher ***CI*** and ***FS*** values. The rest of the non-degraders strains had a weak influence on ***GS*** and ***CI*** albeit no obvious effect on the ***FS***.

Based on the observation of the three parameters, degraders appear to be the fundamental functional unit in the consortia although their effects vary with the strains. *Rhodococcus* has stronger growth-promoting effects on *C. terristeris* evidenced by their association with high ***GS*** values, which could be a result of the efficient single-ring aromatics metabolic systems in this genus (McLeod et al., 2006; Patrauchan et al., 2008; Táncsics et al., 2008). However, due to the same reason, *Rhodococcus* could easily dominate the VOC degrading processes thus outcompeting and shedding the effect of other non-degraders and thus leading to enhanced functional stability (Coyte et al., 2015). Contrary to this, two *Pseudomonas* (degrader A, C) and *Delftia* (degrader D) were considered to have lower performance in supporting algae photosynthesis, evidenced by their frequent presence in low-***GS*** consortia despite many species in these genera having been recognised as strong aromatic hydrocarbon degraders (Kauppi et al., 1998; Shigematsu et al., 2003). Conversely, while these strains may be less efficient in VOC degrading, their reduced competitiveness left niches for the activities of non-degraders which in turn, may have improved the overall consortia performance, as reflected by high ***FS***. Among the 7 non-degraders, *Cupriavidus metallidurans* and *Rhodococcus Sp_3_* seem to be the only two strains affecting the consortia behaviour and thus were very likely to directly participate in the VOCs degradation process due to their frequent presence in consortia of high ***GS*** and ***CI*** value and metabolic capability reported in other studies (Cébron et al., 2008; Alviz-Gazitua et al., 2019, 2022). Other non-degraders including *Pseudomonas syringae*, *Agromyces atrinae*, *Ochrobactrum anthropic* and two *Plantibacter* strains were seen as ‘weak’ bacteria members in the consortia with little effect on any of the parameters regardless of their presence or absence, nor the variation of their combinations.

### 3.6 VOCs degradation

Exemplar consortia aligned with screening results and their VOC degradation performance are listed in Table 3.

**Table 3:** Exemplar consortia and their average daily VOCs degradation within 7 days.

Consortia	Screening parameters			VOCs degradation rate (mg/L)					OD_685_	Growth pattern
	*GS*	*CI*	*FS*	B	T	P	THF	Total		
B14	7.19	2.72	0.62	30.85	32.79	96.54	*3.20	163.39	0.33	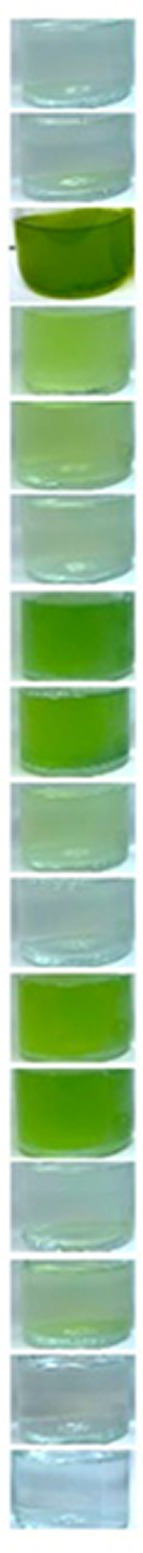
E14	9.86	−0.45	0.19	34.49	37.76	84.28	*25.09	181.61	0.13	
B23	6.86	3.25	0.72	85.49	85.81	91.38	*10.95	273.62	0.91	
E23	12.80	−1.42	0.07	95.72	92.70	93.76	*26.74	308.91	0.53	
B27	3.03	1.25	0.92	20.98	23.02	100	*11.36	157.99	0.32	
E27	10.97	−2.16	−0.03	63.41	65.62	100	*9.17	243.14	0.27	
B123	9.19	6.30	0.84	92.14	87.48	100	*11.97	291.77	0.86	
E123	8.49	1.85	0.45	87.89	87.80	93.92	*12.11	281.71	0.56	
B125	10.15	4.00	0.56	25.03	43.86	94.56	*33.38	196.83	0.45	
E125	10.59	−0.13	0.20	38.46	54.83	97.68	*19.81	210.78	0.28	
B123456	5.23	4.35	0.69	65.26	69.67	91.22	*25.55	251.71	0.84	
E123456	5.13	4.07	0.67	82.52	84.86	91.86	*21.09	280.34	0.68	
B124567	5.41	1.02	0.30	21.69	37.06	96.53	*5.48	160.76	0.36	
E124567	6.31	0.27	0.19	26.90	40.43	98.45	*9.06	174.84	0.37	
CT	0	N/A	N/A	*32.35	*34.09	*5.60	*6.87	*78.92	0.01	
BK	N/A	N/A	N/A	*23.13	*27.23	*3.06	*3.28	*56.70	0.00	

Consortia name indicates the bacterial strains of the consortia following the labels listed in Table 2; All 14 consortia contain microalgae *C. terristeris*. CT: axenic algae control; BK: media-only blank; B: benzene, T: toluene, P: phenol, THF: tetrahydrofuran; Data marked with * are estimated results based on experimental fact, for example, a ‘negative’ VOC concentration indicate zero degradation.

**Figure 5:**
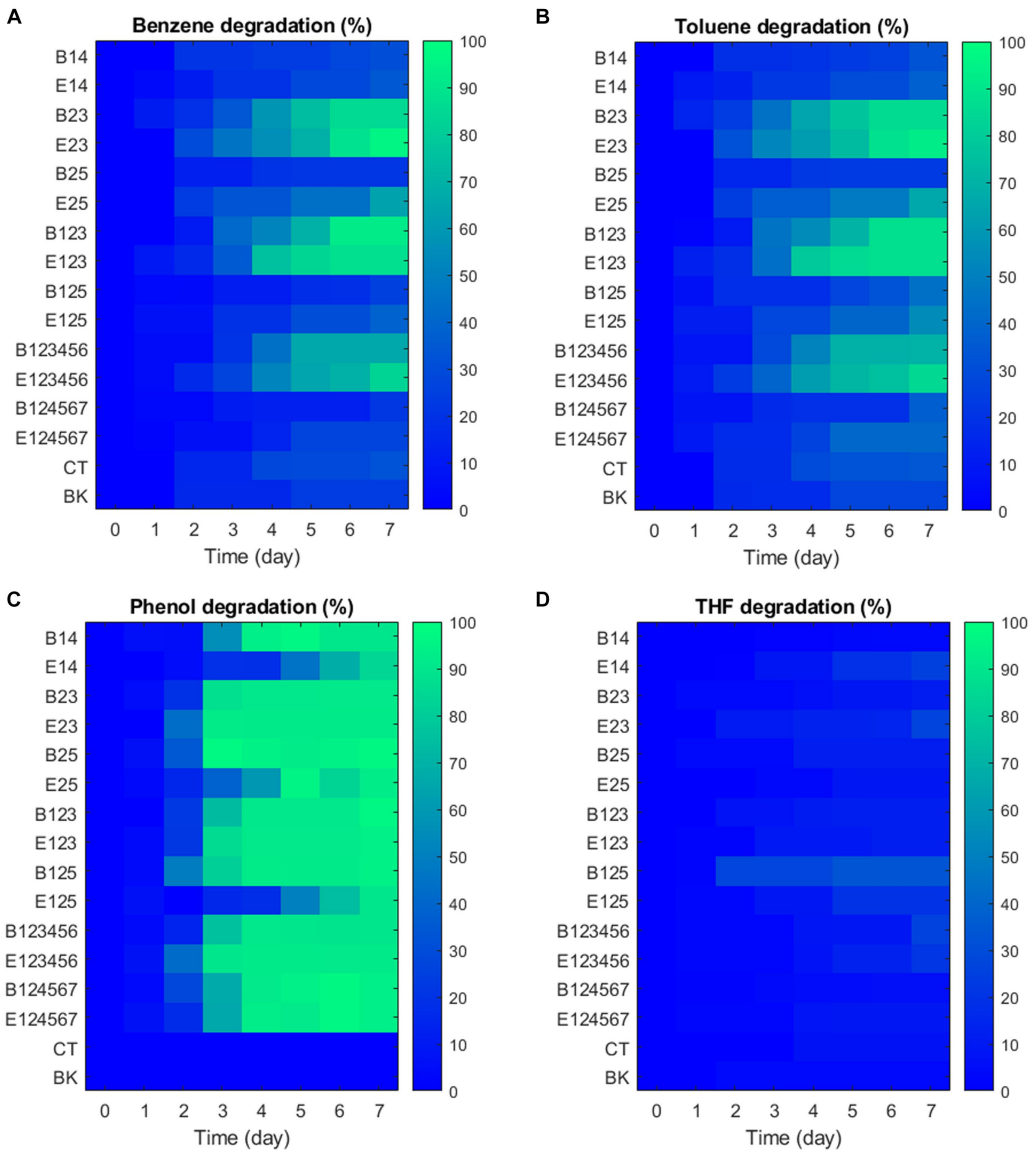
Degradation of VOCs in exemplar consortia. **(A)** Benzene, **(B)** Toluene, **(C)** Phenol, **(D)** THF.

After considering sampling loss and aqueous-gas distributions, GC-FID analysis (Figure 5) confirmed that the VOCs degradation by consortia was primarily biological since no natural degradation was observed in the blank groups despite their high volatility and even light-sensitivities (phenol). Rapid degradation of benzene and toluene occurred mainly between days 2 and 4, with daily degradation rates of 20.61 to 39.86 mg·L^−1^·D^−1^ for benzene and 18.58 to 35.45 mg·L^−1^·D^−1^ for toluene. Consortia B23, E23, B123, E123, B123456, and E123456 showed high benzene removal (63.41–95.71%) and toluene removal (65.62–92.70%) over 7 days. In contrast, other consortia degraded no more than 34.48% of benzene and 40.43% of toluene. Phenol appears to be the most readily degradable VOC due to its relatively central position on aromatic compounds catabolic pathways of associated bacteria (Selvakumaran et al., 2011; Jiang et al., 2015; Alviz-Gazitua et al., 2022). Although phenol degradation was slower in consortia E14, E27 and E125, it was nearly 100% removed within 7 days and served indirectly as the basic carbon source for algae growth in all 14 exemplar consortia. THF showed no obvious sign of degradation by any of the consortia.

Consortia with high VOC removal, including B23, E23, B123, E123, B123456, and E123456, also had high algae biomass content, as indicated by OD_685_ measurements. A strong correlation (*R* = 0.82, *p* < 0.001) was found between total VOC degradation and OD_685_. The performance of consortia in VOC degradation and OD_685_ was influenced by the presence of non-degraders, especially *C. metallidurans*, while no significant difference in VOC degradation was observed between consortia with different degraders (*p* = 0.71).

Surprisingly, no significant correlation was found between total algae biomass and ***GS*** (*R* = −0.18, *p* = 0.54) of these flask-grown exemplar consortia. One possible cause of this deviation is the different sensing mechanisms from which data were generated since the fluorescence signal is measured more sensitively with less interference on the optical pathway caused by suspended bacteria cells (Lakowicz, 2006). Also, larger flask-scale culture volume may influence the growth of algae due to differences in factors such as growth space, nutrient availability and luminance patterns.

Conversely, ***CI*** demonstrated a higher level of efficacy when predicting the algae growth in flask scale exemplar consortia, evidenced by its strong positive correlation with OD_685_ (*R* = 0.73, *p* < 0.001), which could be attributed to its comparative nature, as well as its extended number of observations. A moderate positive correlation lies between OD_685_ and ***FS*** (*R* = 0.58, *p* = 0.003) which, however, reflects the stability of a consortium against the potential influence of additional bacteria as an important reference for the consortia robustness.

### 3.7 Microbial community structure analyses

**Figure 6:**
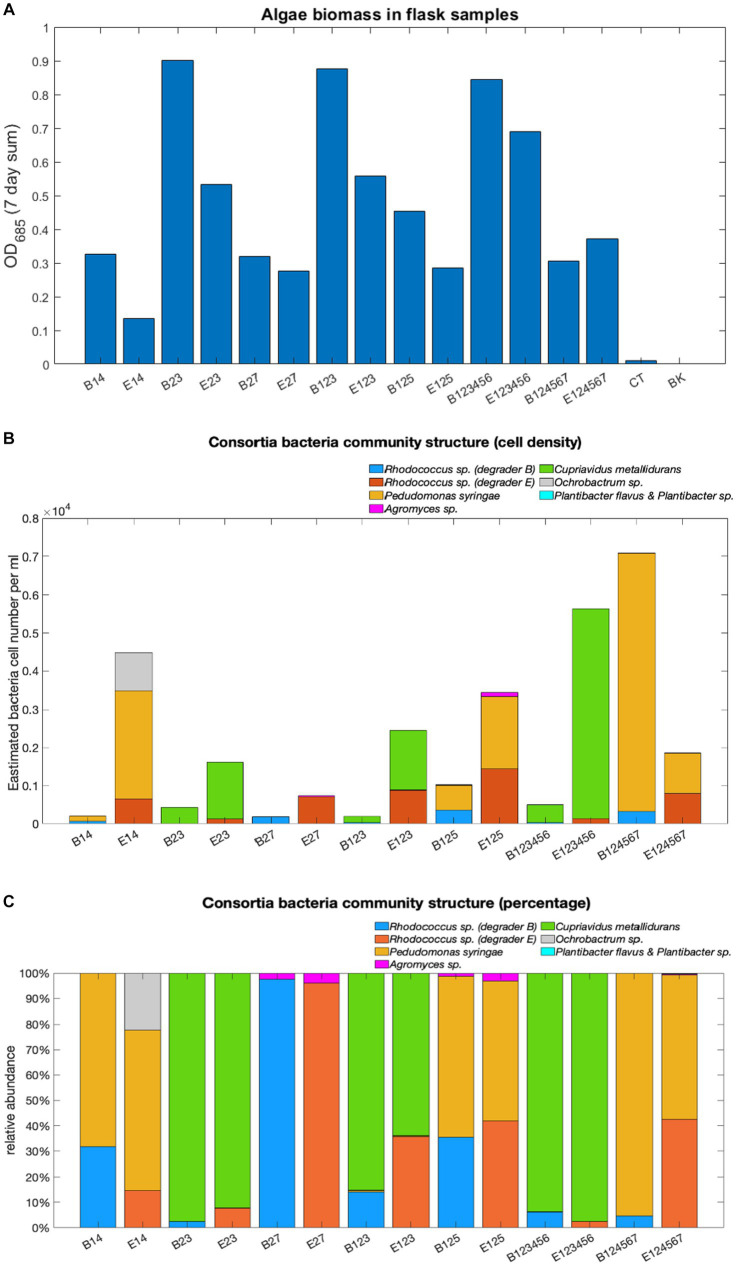
Microbial community structure analyses. Total algae biomass **(A)** calculated as the 7-day sum of OD_685_ values, estimated total bacterial cell density **(B)** and relative abundance **(C)**.

The microbial community structure was profiled in terms of total algae biomass measured as OD685 over a 7-day sum (Figure 6A), total bacterial cell densities (Figure 6B), and the relative abundance of each bacterial strain on day 7 (Figure 6C) within the exemplar consortia.

Both degraders were still detected on day 7, however, their relative abundance varied depending on co-cultured non-degraders. Although both belong to the same genus, the presence of degrader E tends to result in larger overall bacteria populations in the consortia than degrader B (B124567 as an exception). However, little difference was found in the VOCs degradation performance between flask-grown consortia containing the two *Rhodococcus* degraders, suggesting that B is a more competitive strain and may inhibit the growth of other co-cultured bacteria.

Among non-degraders, *C. metallidurans* exhibited the strongest dominance evidenced by its high relative abundance and absence of other co-cultured non-degraders in the associated consortia. The presence of *C. metallidurans* also saw faster degradation of benzene and toluene, suggesting that its aromatic compounds catabolic traits reported in other studies were highly likely to have taken effect in the consortia. ‘Weaker’ non-degraders such as *Agromyces* sp. and *Ochrobactrum* sp. were only found contributing to small proportions of the overall community populations in consortia where *P. syringes* and *C. metallidurans* were absent (except in consortium E14). Neither of the two *Plantibacter* strains (non-degrader 5 and 6) were detected in their associated consortia on Day 7.

Notably, degraders B and E, both from the *Rhodococcus* genus, resulted in distinct community structures. Consortia with degrader B had smaller bacterial populations compared to those with degrader E, suggesting degrader B’s competitive nature, possibly inhibiting co-cultured bacteria growth while favouring algae. This competitive behaviour of degrader B, identified as *R. erythropolis*, might be due to its production of antibiotics effective against various bacteria, including other *Rhodococcus* species (Kitagawa and Tamura, 2008) a trait shared by several *Rhodococcus* species (Kurosawa et al., 2008; Ward et al., 2018). These interactions suggest complexities beyond mere competition for VOCs as a bacterial growth carbon source. However, the limited resolution of 16S rDNA sequencing and the challenges of qPCR analysis in distinguishing between bacteria 7, B, and E in various consortia make it difficult to precisely identify the species involved. Thus, a metabolic profile analysis is recommended for more detailed insights.

Interestingly, consortia with high total VOC degradation had very small bacteria populations and reduced bacteria species diversity, regardless of the number of bacteria strains initially inoculated. This phenomenon was particularly observed with both degrader B and non-degrader 3 (*C. metallidurans*), two competitive strains which support the growth of algae by controlling bacteria populations to small size, which, in turn, enlarged the algae population by making more VOC-originated carbon source photosynthetically available, which reinforces the ‘ecosystem’ model defined in session 3.2. Such consortia were found to have relatively high ***CI*** values and ***FS*** values closer to 1, which further evidence the predictive potential of the two parameters for algae growth and stability.

In summary, consortia exhibiting high VOC degradation performance, particularly those involving *C. metallidurans* and *R. erythropolis*, show great promise in converting VOCs into biomass of *Coelastrella terrestris*. This algae species is beneficial due to its lipid-rich composition (Hodač et al., 2016) thermal resistance (Hu et al., 2013), and carotenoid production capabilities (Rauytanapanit et al., 2019). These characteristics align well with large-scale algae cultivation efforts, particularly those selecting strains for high lipid content for biodiesel production. These consortia would also be a microbial candidate for industrial-scale carbon capture in facilities like power plants, and not only serve as a biological emission control process (Iglina et al., 2022) but also offer the added benefit of VOC remediation.

## 4 Conclusion

This research has successfully demonstrated the bioremediation potential of environmentally isolated individual strains, particularly in the degradation of benzene, toluene, and phenol, and in the efficient conversion of VOCs into algal biomass through well-combined algae-bacteria consortia. The screening method, which utilises total algal chlorophyll content as a proxy, has proven to be highly efficient and rapid in identifying suitable algae-bacteria combinations for VOC degradation. The assessing parameters associated with this method, especially algae growth contribution index (***CI***) and functional stability (***FS***), showed the effectiveness of shortlisting high-performance consortia. Notably, *Rhodococcus erythropolis* and *Cupriavidus metallidurans* emerged as key bacteria in VOC catabolism within these consortia. The screening method provides a benchmark for engineering more stable and effective microbial systems for bioremediation. Moreover, it aids in narrowing the targets for subsequent metabolic or proteomic studies, offering deeper insights into microbial interactions.

This study, however, faced limitations including low-resolution microbial identification and a narrow focus on specific pollutants and strains. Research gaps remain in understanding the real-world application and scalability of industrial VOC waste treatment. Future research should explore a broader range of pollutants and microbes, employ advanced analytical techniques, and focus on practical applications.

## Data availability statement

DNA sequence data presented in the study are deposited in the GenBank repository, accession numbers: PP106129-PP106152, PP106153, and PP106154.

## Author contributions

ZC: Conceptualization, Data curation, Formal analysis, Investigation, Methodology, Software, Visualization, Writing – original draft. EK: Methodology, Validation, Writing – review & editing. JP: Conceptualization, Funding acquisition, Investigation, Project administration, Resources, Supervision, Validation, Writing – review & editing, Methodology.
