# Supplementary material for: Bottom-up construction and screening of algae-bacteria consortia for pollutant biodegradation
Source: Front Microbiol. 2024 Feb 8;15:1349016. doi: 10.3389/fmicb.2024.1349016 (PMC10883772; doi:10.3389/fmicb.2024.1349016)
Supplement: Supplementary file 1 [file Data_Sheet_1.docx]

**Bottom-up Construction and Screening of Algae-bacteria Consortia for Pollutant Biodegradation**

Author: Zongting Cai, Esther Karunakaran, Jagroop Pandhal^*^

**Supplementary material 1**

^*^Author to whom correspondence should be addressed Jagroop Pandhal,

Email: j.pandhal@sheffield.ac.uk

1.VOCs solubility data

| Table S1 Solubility of VOCs compound in water | |
| --- | --- |
| **Compound** | **Solubility in water g/L at 25°C** |
| Benzene | 1.8 |
| Toluene | 0.535  84 |
| Phenol |  |
| Tetrahydrofuran | freely soluble |
| Note:Solubility data source: [*International Programme on Chemical Safety (IPCS)*](https://inchem.org/#/)*.* | |

2 Characteristics of environmental isolates

Following the adaptive culturing, 26 bacteria isolated (included laboratory collection *Pseudomonas putida KT2440*) demonstrated VOCs resistance as evidenced by their observed growths in LB media which contained up to 100 mg/L concentrations for each VOC. The colony morpholohies of these VOCs-resistant bacterial isolates were included in the bacteria collections (Fig.S1). Hypha-forming microorganisms from environmental samples were excluded as they fall outside the scope of this project.

| 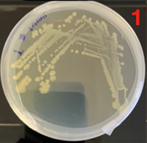 | 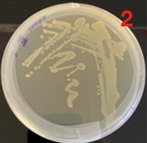 | 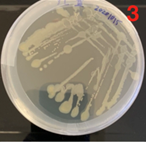 | 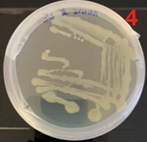 | 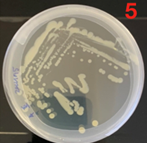 | 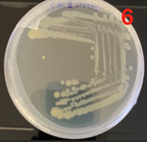 |
| --- | --- | --- | --- | --- | --- |
| 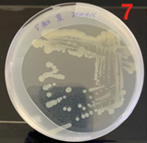 | 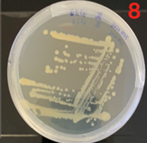 | 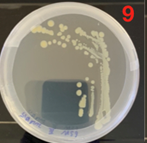 | 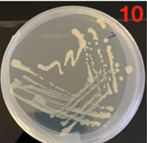 | 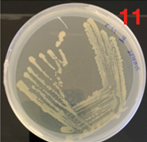 | 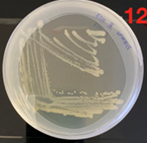 |
| 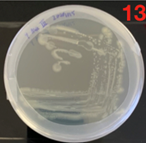 | 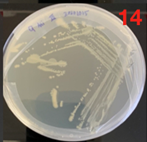 | 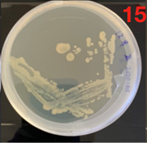 | 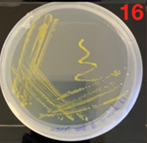 | 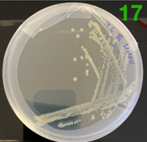 | 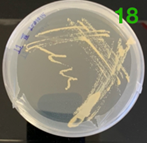 |
| 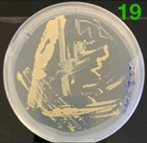 | 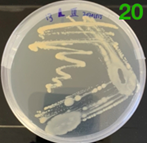 | 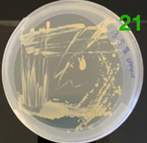 | 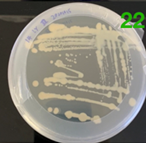 | 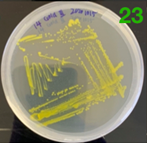 | 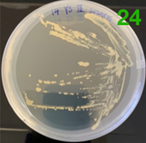 |
| 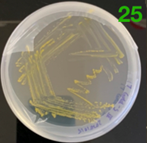 | 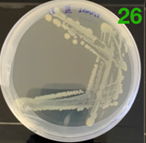 |  |  |  |  |
| Fig.S1: VOC-resistant bacterial isolates. Red numbers indicate strains isolated from mixed colonies, while green represents isolates from a single colony.*Note:The handwritten numbers/letters on the petri dishes in the images are preliminary identifiers used early-stage bacterial isolate differentiation. Please refer to the numerical labels in each image's corner for accurate sample identification. | | | | | |

After autotrophic adaptive culturing, two microalgae strains from soil were isolated. Preliminary identification as two distinct species was based on unique cell morphologies (Fig.S2), temporarily labeled ST, SA. Both strains initially showed resistance to 10 mg/L VOC concentrations and demonstrated effective mixing and suspension in liquid culture.

| A 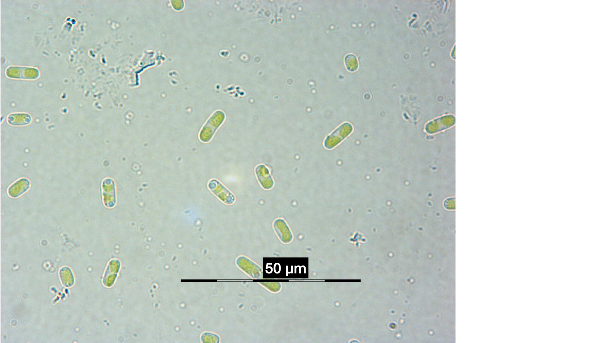 | | | B 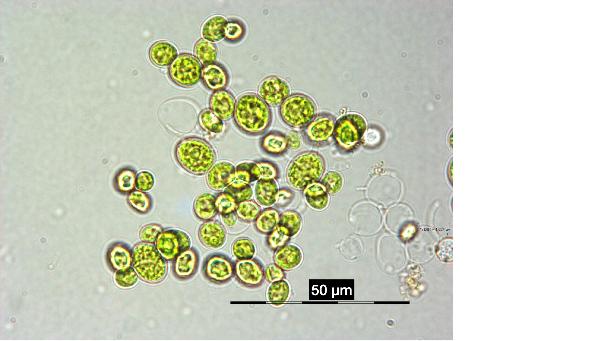 | | |
| --- | --- | --- | --- | --- | --- |
| a1 | a2 | 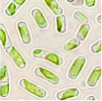a3 | b1 | 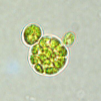b2 | 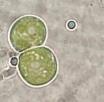b3 |
| Fig.S2 Microscopic images of microalgae isolates A (*ST*), B (*SA*),and their morphology variations during different growth stages: individual cells (a2, b1), cell division (a1,a3;b2). | | | | | |

Carbon-deprived culturing in PBS solution revealed that at least 6 bacteria isolates were able to utilise VOCs as growth substrates (Fig.S3) suggesting their abilities for VOCs degradation. Also, two bacteria isolates (ID:1 and 2) exhibited strong fluorescence under UV light (Fig.S4) which was accidentally discovered during UV sterilisation of the laminar airflow hood.

| **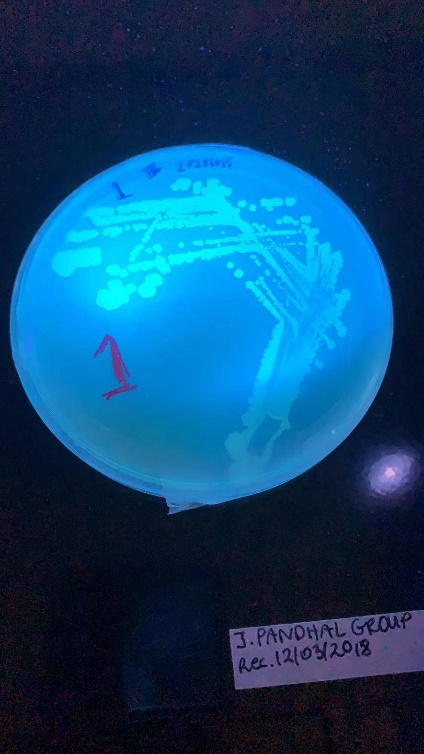A** | **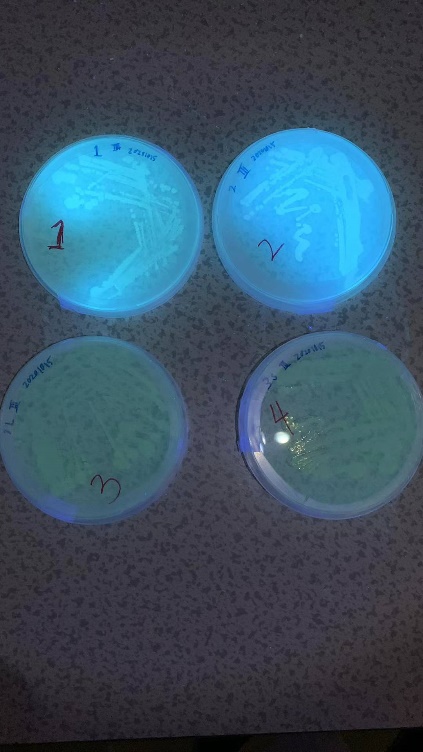B** |
| --- | --- |
| Fig.S3 A**:** Fluorescence in bacterial isolates, indicative of specific colony characteristics which assist in preliminary classification; B**:** comparison between fluorescent and non-fluorescent cultures; culture media: LB agar. | |

| 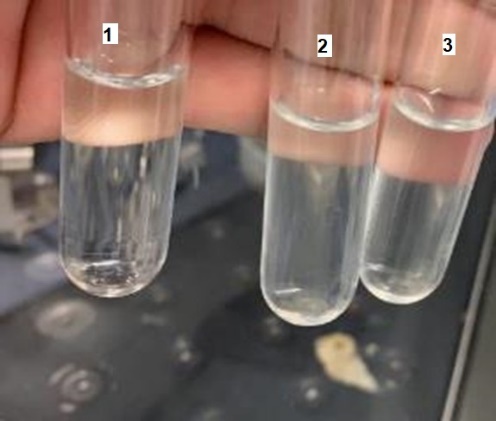 |
| --- |
| Fig.S4 Bacteria isolates grew in PBS solution, using VOCs as only growth substrates. 1: Blank medium, 2 and 3 Bacteria growth resulted in the turbidity of the media. Partial image. |

In an 8-day cultivation period using 3N-BBM+V media, the ST microalgae isolate achieved a growth of 1000$\times$10^4^ cells/mL, while SA reached 45$\times$10^4^ cells/mL. ST had a lower maximum growth rate of 51.5% (day 7) and a shorter lag phase (1 day) compared to SA, which showed a 64.9% growth rate but a longer lag phase (≥ 4 days).

| **A** Cell density |  **B** Optical density |
| --- | --- |
| Fig.S5 Growth curves and lag phases of microalgae isolate ST and SA in VOCs-absent culture condition. Visulaied in both cell density data (A) with dual y-axies and optical density at 680 nm (B). | |

Additionally, the SA strain developed an orange pigment (Fig.S6) after extended culturing (≥14 days), identified as a carotenoid with an absorbance peak between 450 and 470 nm, as per spectrophotometer wavelength scan results (data not shown).

| A | 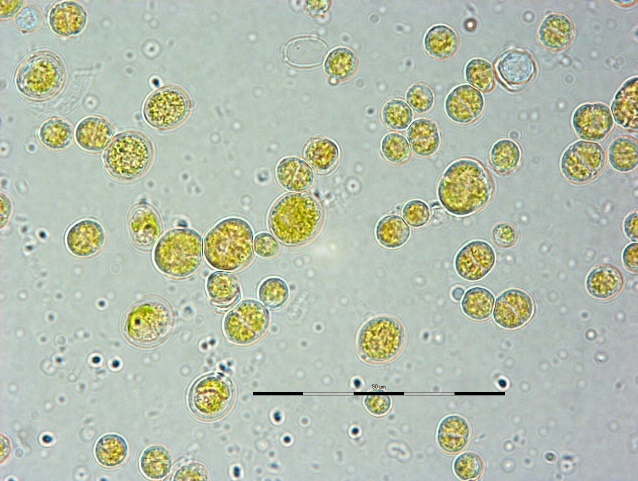B | | 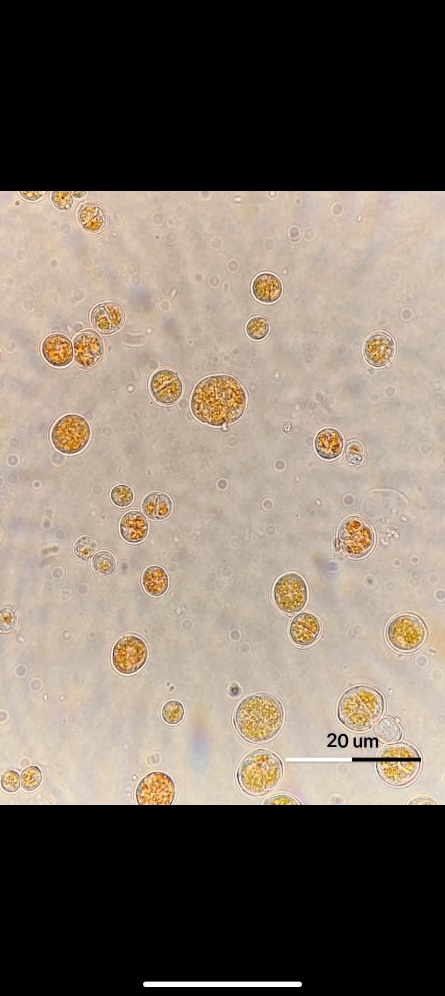C |
| --- | --- | --- | --- |
| 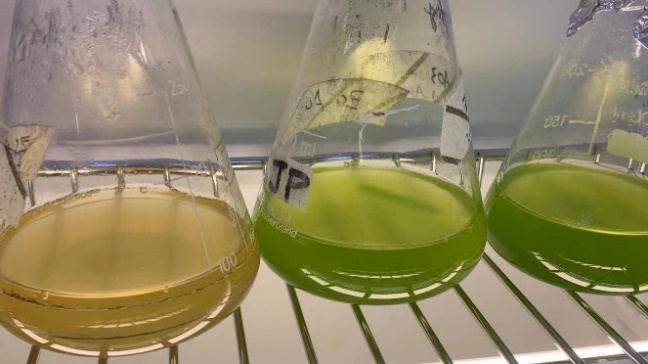D | | 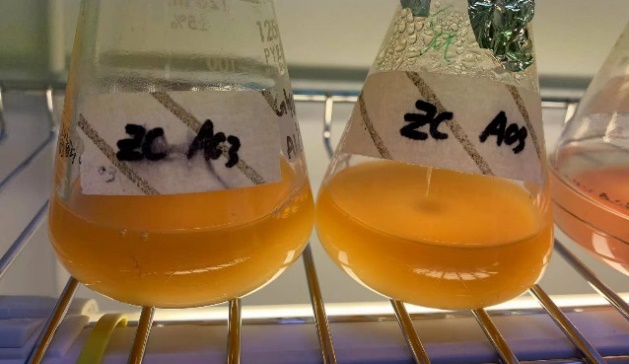E | |
| Fig.S6 Microscopy image of SA strain and colour change over time, A: 0-14 days, B:≥14 days C: preservation stock ≥ 30 days, magnification: $10\times$100; D:14 days; E: ≥ 30 days. | | | |

The VOC resistance experiment showed that both algae strains demonstrated some degree of resistance to VOCs, as evidenced by their growth even under increased VOCs concentrations (Fig.S7). Notably, SA exhibited stronger resistance to VOCs, as suggested by its survival in media where each of the four VOCs were present at a concentration of 200 mg/L. Moreover, the growth of SA was consistently higher than that of ST cells under these conditions. This suggests that the SA strain is a more suitable algae candidate for consortia for VOCs biodegradation.

| 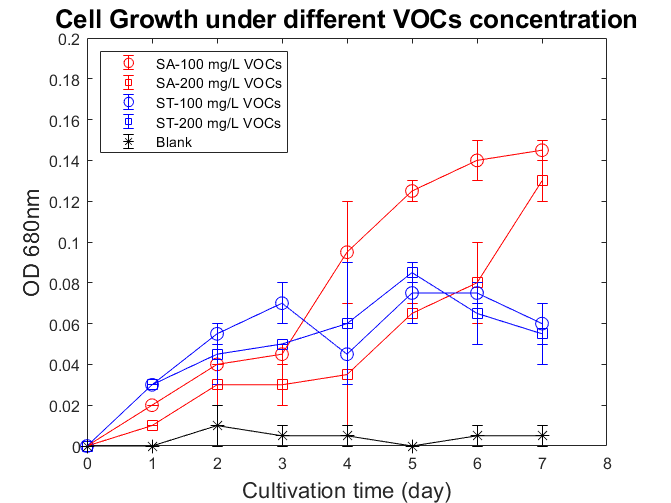 |
| --- |
| Fig.S7 Optical densities at 680 nm of algae isolate ST and SA grown in 3N-BBM+V media containing different concentrations of VOCs. |

3.PCR program setting, and primer sequence for environmental isolate identification

| Table S2 Primer selection and PCR program setting | | | | | | | |
| --- | --- | --- | --- | --- | --- | --- | --- |
| **Template DNA** | **Primer** | | | **Amp. length** | **Program settings** | **Reaction Volume** | **Reference** |
|  | **Name** | **Target region(s)** | **Sequence (5’-3’)** |  |  |  |  |
| **Bacteria rDNA** | 515 (F) | 16s | GTGCCAGCMGCCGCGGTAA | 440 bp | **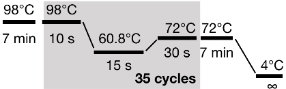** | 50 μl | (Rudi et al., 1997; Turner et al., 1999) |
|  | CD (R) |  | CTTGTGCGGGCCCCCGTCAATTC |  |  |  |  |
|  | 357 (F) | 16s | CTCCTACGGGAGGCAGCAG | 740 bp | **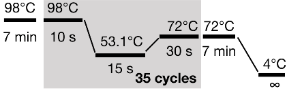** | 50 μl | (Turner et al., 1999) |
|  | 1100 (R) |  | AGGGTTGCGCTCGTTG |  |  |  |  |
| **Algae**  **rDNA** | 18s-SS3 (F) | 18s | GGTGATCCTGCCAGTAGTCATATGCTTG | 1800 bp | **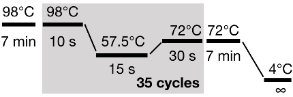** | 50 μl | (Khaw et al., 2020; Matsumoto et al., 2010) |
|  | 18s-SS5(R) |  | GATCCTTCCGCAGGTTCACCTACGGAAACC |  |  |  |  |
|  | ITS_5.8 (F) | ITS1-5.8s-ITS2 | GAAGTCGTAACAAGGTTTCC | 800 bp | **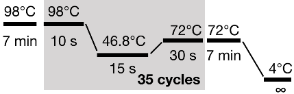** | 50 μl | (Timmins et al., 2009) |
|  | ITS_5.8 (R) |  | TCCTGGTTAGTTTCTTTTCC |  |  |  |  |
|  | PA (F) | 18s | AACCTGGTTGATCCTGCCAG | 500 bp | **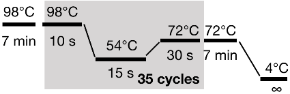** | 50 μl | (Alemzadeh et al., 2014) |
|  | SSU-inR1 (R) |  | CACCAGACTTGCCCTCCA |  |  |  |  |
|  | P45 (F) | 18s | ACCTGGTTGATCCTGCCAGT | 550 bp | 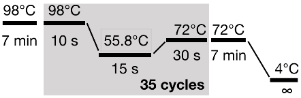 | 50 μl | (Duong et al., 2012) |
|  | P47(R) |  | TCTCAGGCTCCCTCTCCGGA |  |  |  |  |

4 Identification of environmental isolates

DNA sequences were NCBI BLAST-analysed using Nucleotide Collections (Standard databases) and the best match results suggested that the 26 bacteria isolates belong to 10 genera while the two sequenced microalgae were identified as *Deuterostichococcus epilithicus* and *Coelastrella terrestris*, respectively (Table S3).

| Table S3 Microorganism identification results | | | |
| --- | --- | --- | --- |
| Bateria/microalgae label | NCBI BLAST results | Match | Sequence accession number(s) |
| 1 | *Pseudomonas fluorescens* | 100% | PP106129 |
| 2 | *Pseudomonas sp.* | 100% | PP106130 |
| 3 | *Pseudomonas syringae* | 100% | PP106131 |
| 4 | *Stenotrophomonas sp. L18Cg* | 100% | PP106132 |
| 5 | *Stenotrophomonas sp.* | 100% | PP106133 |
| 6 | *Pseudomonas laurentiana* | 100% | PP106134 |
| 7 | *Stenotrophomonas sp. L18Cg* | 100% | PP106135 |
| 8 | *Stenotrophomonas rhizophila* | 100% | PP106136 |
| 9 | *Stenotrophomonas sp. B3_22* | 100% | PP106137 |
| 10 | *Achromobacter sp. strain Bbqt9* | 97.92% | PP106138 |
| 11 | *Pseudomonas sp. strain SeaQual_P_B845W* | 100% | PP106139 |
| 12 | *Pseudomonas laurentiana* | 100% | PP106140 |
| 13 | *Delftia tsuruhatensis* | 100% | PP106141 |
| 14 | *Delftia tsuruhatensis* | 100% | PP106142 |
| 15 | *Cupriavidus metallidurans* | 100% | PP106143 |
| 16 | *Plantibacter flavus* | 100% | PP106144 |
| 17 | *Rhodococcus sp.* | 100% | PP106145 |
| 18 | *Rhodococcus sp.* | 100% | PP106146 |
| 19 | *Rhodococcus erythropolis* | 100% | PP106147 |
| 20 | *Rhodococcus sp.* | 100% | PP106148 |
| 21 | *Rhodococcus sp.* | 100% | PP106149 |
| 22 | *Ochrobactrum anthropi* | 100% | PP106150 |
| 23 | *Agromyces atrinae strain P27 (T)* | 100% | PP106151 |
| 24 | *Rhodococcus sp.* | 100% | PP106152 |
| 25 | *Plantibacter sp.* (not same as 16) | 100% | PP106153 |
| 26 | *Pseudomonas putida KT2440* | N/A* | N/A* |
| ST | *Deuterostichococcus epilithicus (Stichococcus sp.)* | 99% | PP106154 |
| SA | *Coelastrella terrestris* | 99.8% | PP106155 |
| *Note: Bacteria 26 is a known strain of *Pseudomonas putida KT2440* obtained from laboratory collection and was not sequenced. | | | |

5. Fluorescent data validation

Chlorophyll fluorescence (Ex 390 nm, Em 690) data collected by the plate reader showed strong linear correlations to dilution factors in the three calibrating samples (Fig.S8). While the y-intercepts of the three samples differed due to variations in initial cell densities and chlorophyll content in different algae strains, the gradients (1-degree polynomial) of three sets of data were close to 0.693, the natural logarithm of 2, which closely aligned with the feature of two-fold dilutions. This evidenced the high accuracy in the quantification of algae biomass by directly measuring chlorophyll fluorescence, despite the presence of bacteria which could potentially interfere with fluorescence signals.

| **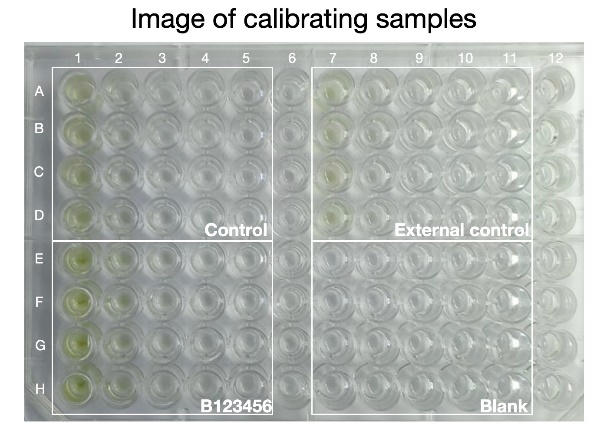a** | 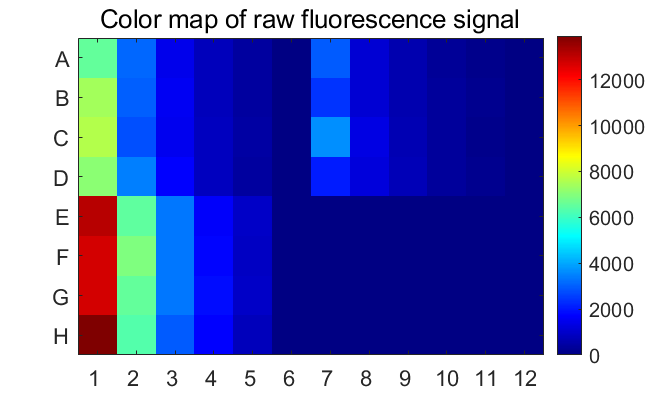**b** |
| --- | --- |
| 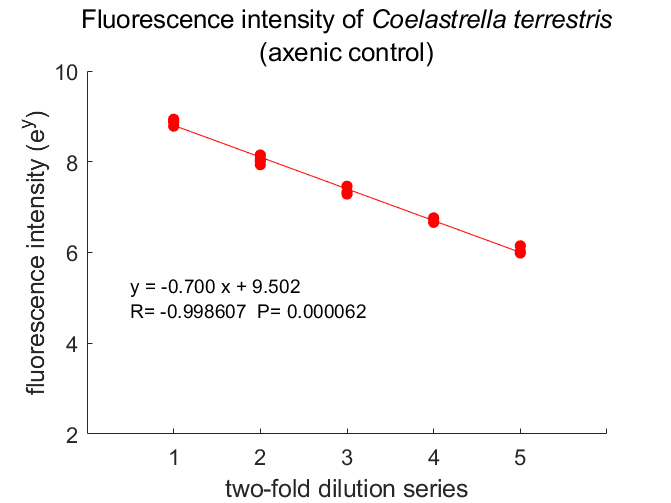**c** | 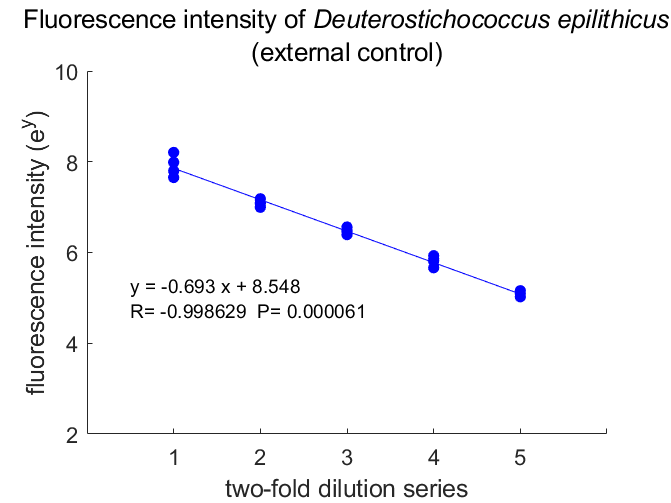**d** |
| **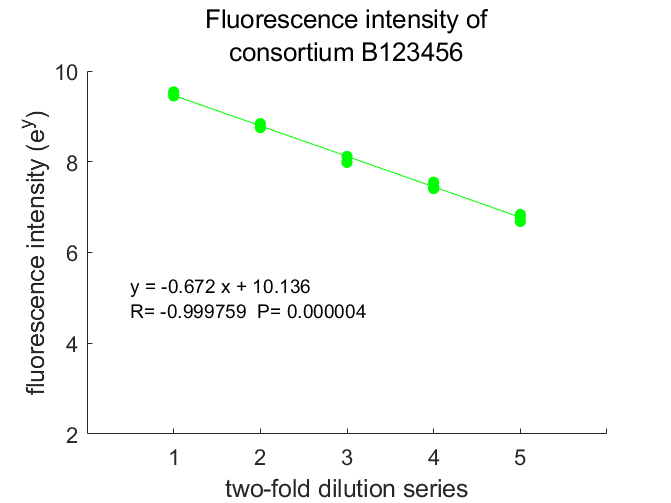e** | **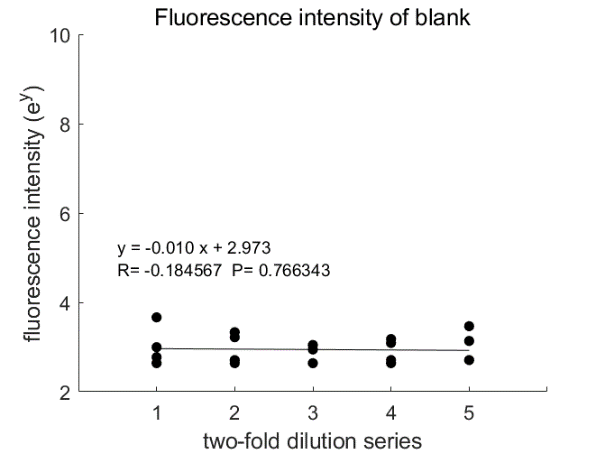f** |
| Fig.S8 Linear fitting of the fluorescence signal under natural logarithm and algae cell density (**a** and **b**) in axenic control *Coelastrella terrestris* (**c**), external control *Deuterostichococcus epilithicus* (previously known as *Stichococcus sp.*) (**d**), and exemplar algae-bacteria consortium (**e**) and blank (**f**). | |

6. VOCs concentration determination

6.1 Henrry’s Law correction

Notably, VOC concentration data obtained from GC analysis may not accurately reflect the true VOC quantities in liquid samples, due to the variation of the extraction efficiencies of different VOC compounds transmitting from the medium into the organic solvent. However, the extraction efficiency, also known as the Partition Coefficient (*P*), is inherently dependent on the properties of the solvent system and the analyte to be extracted (Moldoveanu and David, 2015a). While data of Partitioning Coefficient in water/DCM systems remain limited, Octanol-Water is a well-established for solvent systems wherein the Octanol-Water Partition Coefficients ($P_{ow}$) of different analytes are available. Also, linear dependence exists between the logarithm of $P_{ow}$ for a solute and the logarithm of Partition Coefficient for the same solute in an similar solvent systems (Moldoveanu and David, 2015b; Amézqueta et al., 2020). It is therefore reasonable to hypotheses that for a specific analyte in this study, the extraction efficiency is a constant and is equal to the Partitioning Coefficient in water/DCM systems which can be obtained by:

| $P_{H_{2}O/DCM}=\frac{{C_{aq}^{'}}_{0}}{{C_{aq}}_{0}}$ | Eq.S1 |
| --- | --- |

Where ${C_{aq}^{'}}_{0}$ and ${C_{aq}}_{0}$ are concentrations of a specific VOC detected by GC analysis and its initial concentration in the medium (i.e. 100 mg/L), respectively.

Assuming that all samples taken in this experiment were at partitioning equilibrium, for day $i$ the true quantity of this specific VOC can be estimated via $P_{H_{2}O/DCM}$using the following equation:

| ${C_{aq}}_{i}={2\times C_{aq}^{'}}_{i}{\times P}_{H_{2}O/DCM}$ | Eq.S2 |
| --- | --- |
|  |  |

In addition, due to the volatile nature of the VOCs involved in this study, their distribution in aqueous medium and headspace air was considered. Since the volume ratio of DCM to medium in the extraction is 0.5:1 mL, ${C_{aq}}_{i}$is supposed to time 2.

Using ${C_{aq}}_{i}$ data, the quantity of analyte in the medium can be calculated via Eq.S3

| ${N_{aq}}_{i}=\frac{{C_{aq}}_{i}\times{V_{aq}}_{i}\times6.022\times{10}^{23} {mol}^{-1}}{M}$ | Eq.S3 |
| --- | --- |

Where ${N_{aq}}_{i}$ is the quantity of analyte in the aqueous phase, $M$ is the molecular weight of the analyte VOC compound (g·mol^-1^). ${V_{aq}}_{i}$ Is the volumes of the aqueous medium on day $i$*,*

The distribution of the volatile analyte in water and headspace air obeys Henry’s Law (Schwardt et al., 2021) using the following equation:

| $P_{i}=\frac{{C_{aq}}_{i}}{k_{H}}$ | Eq.S4 |
| --- | --- |

where $P_{i}$ is the partial pressure of the analyte in the gas phase on day $i$ ; $k_{H}$ is the Henry's Law constant (mol·m^-^³·Pa^-1^) of the analyte (when water is used as solvent) under certain atmospheric pressure and temperature.

The amount of analyte in the headspace air can be determined using Ideal Gas Law via the following equation

| ${N_{gs}}_{i}=\frac{P_{i}\times{V_{gs}}_{i}}{k_{B}\times T}$ | Eq.S4 |
| --- | --- |

Where the ${N_{gs}}_{i}$and ${V_{gs}}_{i}$ are the amount of analyte in the gas phase and the volume of headspace air. $k_{B}$is the Boltzmann constant ($k_{B}=1.380649\times{10}^{-23} J/K$). $T$ demotes the kelvin temperature ($K$).

Given that the incubation bottles were gas-tight and were seen as closed systems, the mass balance principle can be applied using:

| $N_{i}={N_{aq}}_{i}+{N_{gs}}_{i}$ | Eq.S5 |
| --- | --- |

In addition, the changes in liquid-headspace volume ratio as a result of daily sampling are expected to alter the VOCs distributions between the aqueous and gas phases. Considering this factor, the total VOCs quantities within the incubator bottle were subject to correction using the following equation:

| $\left\{ \begin{aligned} {Dg}_{i}=N_{i}-N_{i+1}-{N_{loss}}_{i} \\ {N_{loss}}_{i}=V_{sample}\times{C_{aq}}_{i} \\ {V_{aq}}_{i+1}={V_{aq}}_{i}-V_{sample} \end{aligned} \right.$ | Eq.S6 |
| --- | --- |

where ${Dg}_{i}$ is the amount (mole) of a specific VOC analyte being degraded biologically on day $i$ , $N_{i}$ and $N_{i+1}$are its amount in the entire incubation bottle on day $i$ and $i+1$. ${C_{aq}}_{i}$ is the concentration of analyte in the aqueous medium determined by GC analyses. ${V_{aq}}_{i+1}$is the volumes of the aqueous medium on day $i$ and $i+1$. $V_{sample}$ refers to the volume of liquid samples taken daily. This correction was also validated using a computational simulation. The Henry’s Law constants for the four VOCs adopted in this study are listed in Table S4.

| Table S4 Henry's Law Constants of VOCs | | | |
| --- | --- | --- | --- |
| **VOC Name** | $\boldsymbol{k}_{\boldsymbol{H}}\boldsymbol{(}\frac{\boldsymbol{mol}}{\boldsymbol{m}^{\boldsymbol{3}}\boldsymbol{Pa}}\boldsymbol{)}$ | **reference** | $\boldsymbol{k}_{\boldsymbol{H}}$ **in this study** |
| Benzene | 1.7$\times$10^-3^ | (Kim and Kim, 2014) | 1.75$\times$10^-3^ |
|  | 1.8$\times$10^-3^ | (Hiatt, 2013)) |  |
|  | 1.7$\times$10^-3^ | (Sieg et al., 2009) |  |
|  | 1.8$\times$10^-3^ | (Jian, 2008) |  |
|  |  |  |  |
| Toluene | 1.5$\times$10^-3^ | (Kutsuna and Kaneyasu, 2021) | 1.55$\times$10^-3^ |
|  | 1.5$\times$10^-3^ | (Kim and Kim, 2014) |  |
|  | 1.7$\times$10^-3^ | (Lee et al., 2013) |  |
|  | 1.5$\times$10^-3^ | (Kish et al., 2013) |  |
|  |  |  |  |
| Phenol | 3.0 | (Harrison et al., 2002) | 3.2 |
|  | 4.2 | (Duchowicz et al., 2020) |  |
|  | 3.6 | (Wang et al., 2017) |  |
|  | 2.0 | (Mackay et al., 2006) |  |
|  |  |  |  |
| THF | 2.2$\times$10^-1^ | (Signer et al., 1969) | 1.45$\times$10^-1^ |
|  | 1.4$\times$10^-1^ | (Cabani et al., 1971) |  |
|  | 1.1$\times$10^-1^ | (Hilal et al., 2008) |  |
|  | 1.1$\times$10^-1^ | (Abraham et al., 1990) |  |
| Note: Henry’s Law constants listed above are under 298.15 K (25**°C**). Constants adopted in this study derive from average values of the most recent studies with experimental evidence whenever available. | | | |

6.2 VOCs loss by sampling

The computational result of VOCs loss due to sampling, as presented in Fig.S9, which indicates that all four VOCs experienced a continuous sampling loss, particularly in the blank (BK) and control (CT) bottles, which exhibit a near-linear loss. This VOCs loss due to sampling accounts for less than 14% of the total VOCs in the entire incubation bottles. Phenol (Fig.S9 c) and THF (Fig.S9 d), which have a higher affinity for water, underwent more pronounced sampling loss. However, consortia with major aqueous VOCs concentration reductions (manuscript) demonstrated a decelerated VOCs sampling loss ranging from 2-6% depending on the types of VOCs. It is evident that consortia with a faster reduction of VOCs retain fewer VOCs in the bottles, consequently resulting in even lower VOCs loss through sampling. Therefore, any significant VOCs concentration reduction observed in GC-FID analysis is likely due to biological degradation rather than sampling loss.

| 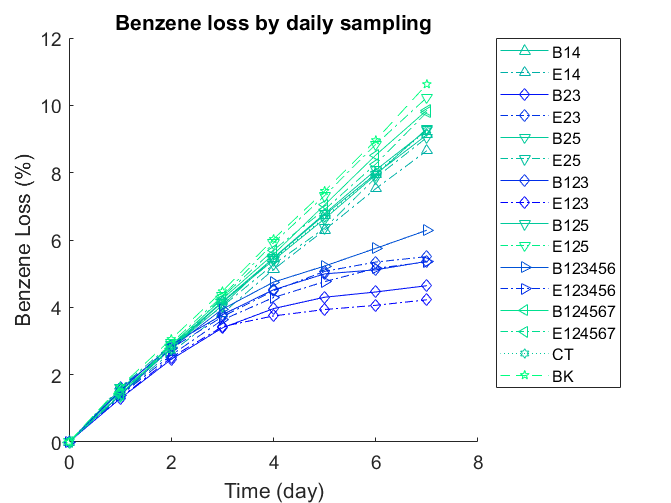a | 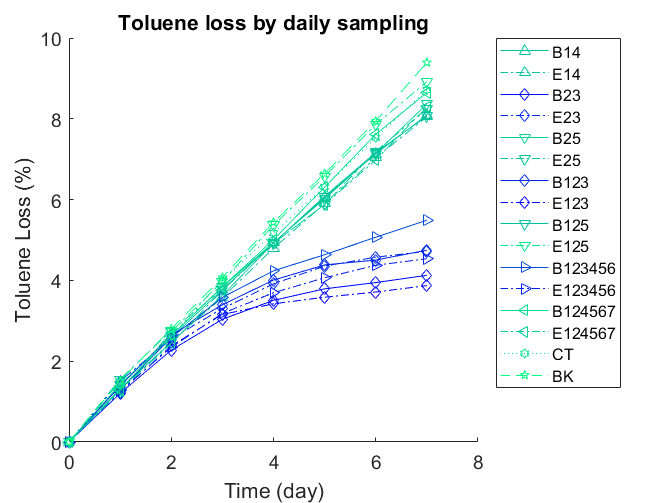b |
| --- | --- |
| 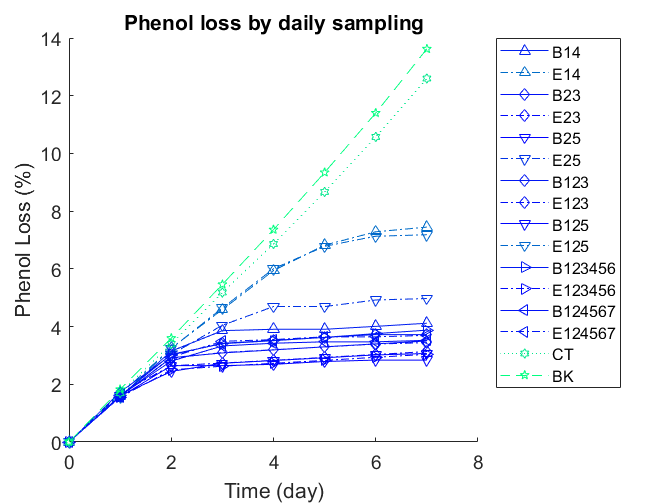c | 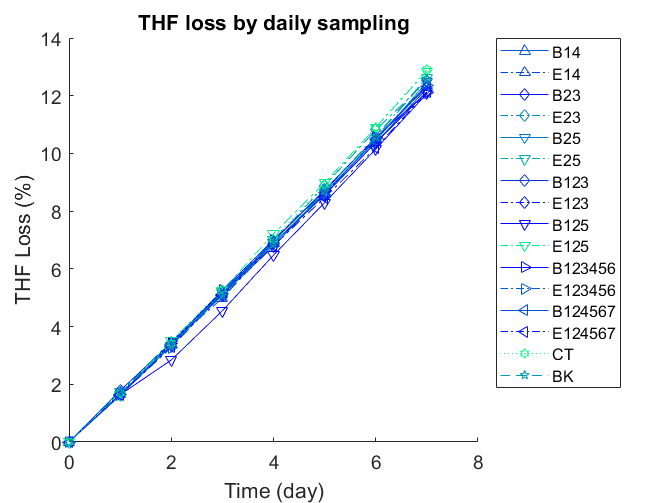d |
| Fig.S9 VOCs sampling loss in each consortium (calculated via Henry’s Law); a: Benzene, b: Toluene, c: Phenol and d: THF. | |

7. 96-well plates layout for screening method

| Table S5 (part 1). 96-well plates layout for screening | | | | | | | | | | | | | | | | | | | | | | | |
| --- | --- | --- | --- | --- | --- | --- | --- | --- | --- | --- | --- | --- | --- | --- | --- | --- | --- | --- | --- | --- | --- | --- | --- |
| S2 and S2 copy | | | | | | | | | | | | S3 I / S3 I copy | | | | | | | | | | | |
| A1 | A2 | A3 | A4 | B1 | B2 | B3 | B4 | C1 | C2 | C3 | C4 | A12 | A13 | A14 | A15 | B12 | B13 | B14 | B15 | C12 | C13 | C14 | C15 |
| A5 | A6 | A7 | A | B5 | B6 | B7 | B | C5 | C6 | C7 | B | A16 | A17 | A23 | A24 | B16 | B17 | B23 | B24 | C16 | C17 | C23 | C24 |
| D1 | D2 | D3 | D4 | E1 | E2 | E3 | E4 | F1 | F2 | F3 | F4 | A25 | A26 | A27 | A34 | B25 | B26 | B27 | B34 | C25 | C26 | C27 | C34 |
| D5 | D6 | D7 | B | E5 | E6 | E7 | B | F5 | F6 | F7 | B | A35 | A36 | A37 | A45 | B35 | B36 | B37 | B45 | C35 | C36 | C37 | C45 |
| A1 | A2 | A3 | A4 | B1 | B2 | B3 | B4 | C1 | C2 | C3 | C4 | A46 | A47 | A56 | A57 | B46 | B47 | A56 | B57 | C46 | C47 | C56 | C57 |
| A5 | A6 | A7 | B | B5 | B6 | B7 | A | C5 | C6 | C7 | B | A67 | A | A | A | B67 | B | B | B | C67 | C | C | C |
| D1 | D2 | D3 | D4 | E1 | E2 | E3 | E4 | F1 | F2 | F3 | F4 | A | A | A | A | B | B | B | B | C | C | C | C |
| D5 | D6 | D7 | B | E5 | E6 | E7 | B | F5 | F6 | F7 | B | A | A | A | A | B | B | B | B | C | C | C | C |
| S3 II / S3 II copy | | | | | | | | | | | | S4 I / S4 I copy | | | | | | | | | | | |
| D12 | D13 | D14 | D15 | E12 | E13 | E14 | E15 | F12 | F13 | F14 | F15 | A123 | A124 | A125 | A126 | A127 | A135 | A134 | A136 | A137 | A145 | A146 | A147 |
| D16 | D17 | D23 | D24 | E16 | E17 | E23 | E24 | F16 | F17 | F23 | F24 | A156 | A157 | A167 | A234 | A235 | A236 | A237 | A245 | A246 | A247 | A256 | A257 |
| D25 | D26 | D27 | D34 | E25 | E26 | E27 | E34 | F25 | F26 | F27 | F34 | A267 | A345 | A346 | A347 | A356 | A357 | A367 | A456 | A457 | A467 | A567 | A |
| D35 | D36 | D37 | D45 | E35 | E36 | E37 | E45 | F35 | F36 | F37 | F45 | B123 | B124 | B125 | B126 | B127 | B134 | B135 | B136 | B137 | B145 | B146 | B147 |
| D46 | D47 | D56 | D57 | E46 | E47 | A56 | E57 | F46 | F47 | F56 | F57 | B156 | B157 | B167 | B234 | B235 | B236 | B237 | B245 | B246 | B247 | B256 | B257 |
| D67 | D | D | D | E67 | E | E | E | F67 | F | F | F | B267 | B345 | B346 | B347 | B356 | B357 | B367 | B456 | B457 | B467 | B567 | B |
| D | D | D | D | E | E | E | E | F | F | F | F | C123 | C124 | C125 | C126 | C127 | C134 | C135 | C136 | C137 | C145 | C146 | C147 |
| D | D | D | D | E | E | E | E | F | F | F | F | C156 | C157 | C167 | C234 | C235 | C236 | C237 | C245 | C246 | C247 | C256 | C257 |

.

| Table S5 (part 2). 96-well plates layout for screening | | | | | | | | | | | | | | | | | | | | | | | |
| --- | --- | --- | --- | --- | --- | --- | --- | --- | --- | --- | --- | --- | --- | --- | --- | --- | --- | --- | --- | --- | --- | --- | --- |
| S4II/S4II copy | | | | | | | | | | | | S4 III and S4 III copy | | | | | | | | | | | |
| C267 | C345 | C346 | C347 | C356 | C357 | C367 | C456 | C457 | C467 | C567 | C | F156 | F157 | F167 | F234 | F235 | F236 | F237 | F245 | F246 | F247 | F256 | F257 |
| D123 | D124 | D125 | D126 | D127 | D134 | D135 | D136 | D137 | D145 | D146 | D147 | F267 | F345 | F346 | F347 | F356 | F357 | F367 | F456 | F457 | F467 | F567 | F |
| D156 | D157 | D167 | D234 | D235 | D236 | D237 | D245 | D246 | D247 | D256 | D257 | CT | CT | CT | CT | CT | CT | CT | CT | CT | CT | CT | CT |
| D267 | D345 | D346 | D347 | D356 | D357 | D367 | D456 | D457 | D467 | D567 | D | CT | CT | CT | CT | CT | CT | CT | CT | CT | CT | CT | CT |
| E123 | E124 | E125 | E126 | E127 | E134 | E135 | E136 | E137 | E145 | E146 | E147 | CT | CT | CT | CT | CT | CT | CT | CT | CT | CT | CT | CT |
| E156 | E157 | E167 | E234 | E235 | E236 | E237 | E245 | E246 | E247 | E256 | E257 | CT | CT | CT | CT | CT | CT | CT | CT | CT | CT | CT | CT |
| E267 | E345 | E346 | E347 | E356 | E357 | E367 | E456 | E457 | E467 | E567 | E | F267 | F345 | F346 | F347 | F356 | F357 | F367 | F456 | F457 | F467 | F567 | F |
| F123 | F124 | F125 | F126 | F127 | F134 | F135 | F136 | F137 | F145 | F146 | F147 | F156 | F157 | F167 | F234 | F235 | F236 | F237 | F245 | F246 | F247 | F256 | F257 |
| S5 I /S5 I copy | | | | | | | | | | | | S5 II / S5 II copy | | | | | | | | | | | |
| A1234 | A1235 | A1236 | A1237 | A1245 | A1246 | A1247 | A1256 | A1257 | A1267 | A1345 | A1346 | C2357 | C2367 | C2456 | C2457 | C2467 | C2567 | C3456 | C3457 | C3467 | C3576 | C4567 | C |
| A1347 | A1356 | A1357 | A1367 | A1456 | A1457 | A1467 | A1567 | A2345 | A2346 | A2347 | A2356 | D1234 | D1235 | D1236 | D1237 | D1245 | D1246 | D1247 | D1256 | D1257 | D1267 | D1345 | D1346 |
| A2357 | A2367 | A2456 | A2457 | A2467 | A2567 | A3456 | A3457 | A3467 | A3576 | A4567 | A | D1347 | D1356 | D1357 | D1367 | D1456 | D1457 | D1467 | D1567 | D2345 | D2346 | D2347 | D2356 |
| B1234 | B1235 | B1236 | B1237 | B1245 | B1246 | B1247 | B1256 | B1257 | B1267 | B1345 | B1346 | D2357 | D2367 | D2456 | D2457 | D2467 | D2567 | D3456 | D3457 | D3467 | D3576 | D4567 | D |
| B1347 | B1356 | B1357 | B1367 | B1456 | B1457 | B1467 | B1567 | B2345 | B2346 | B2347 | B2356 | E1234 | E1235 | E1236 | E1237 | E1245 | E1246 | E1247 | E1256 | E1257 | E1267 | E1345 | E1346 |
| B2357 | B2367 | B2456 | B2457 | B2467 | B2567 | B3456 | B3457 | B3467 | B3576 | B4567 | B | E1347 | E1356 | E1357 | E1367 | E1456 | E1457 | E1467 | E1567 | E2345 | E2346 | E2347 | E2356 |
| C1234 | C1235 | C1236 | C1237 | C1245 | C1246 | C1247 | C1256 | C1257 | C1267 | C1345 | C1346 | E2357 | E2367 | E2456 | E2457 | E2467 | E2567 | E3456 | E3457 | E3467 | E3576 | E4567 | E |
| C1347 | C1356 | C1357 | C1367 | C1456 | C1457 | C1467 | C1567 | C2345 | C2346 | C2347 | C2356 | F1234 | F1235 | F1236 | F1237 | F1245 | F1246 | F1247 | F1256 | F1257 | F1267 | F1345 | F1346 |

| Table S5 (part 3). 96-well plates layout for screening | | | | | | | | | | | | | | | | | | | | | | | |
| --- | --- | --- | --- | --- | --- | --- | --- | --- | --- | --- | --- | --- | --- | --- | --- | --- | --- | --- | --- | --- | --- | --- | --- |
| S5 III and S5 III copy | | | | | | | | | | | | S6 I / S6 I copy | | | | | | | | | | | |
| F1347 | F1356 | F1357 | F1367 | F1456 | F1457 | F1467 | F1567 | F2345 | F2346 | F2347 | F2356 | A12345 | A12346 | A12347 | A12356 | A12357 | A12367 | A12456 | A12457 | A12467 | A12567 | A13456 | A13457 |
| F2357 | F2367 | F2456 | F2457 | F2467 | F2567 | F3456 | F3457 | F3467 | F3576 | F4567 | F | A13467 | A13567 | A14567 | A23456 | A23457 | A23467 | A23567 | A24567 | A34567 | A | A | A |
| CT | CT | CT | CT | CT | CT | CT | CT | CT | CT | CT | CT | B12345 | B12346 | B12347 | B12356 | B12357 | B12367 | B12456 | B12457 | B12467 | B12567 | B13456 | B13457 |
| CT | CT | CT | CT | CT | CT | CT | CT | CT | CT | CT | CT | B13467 | B13567 | B14567 | B23456 | B23457 | B23467 | B23567 | B24567 | B34567 | B | B | B |
| CT | CT | CT | CT | CT | CT | CT | CT | CT | CT | CT | CT | C12345 | C12346 | C12347 | C12356 | C12357 | C12367 | C12456 | C12457 | C12467 | C12567 | C13456 | C13457 |
| CT | CT | CT | CT | CT | CT | CT | CT | CT | CT | CT | CT | C13467 | C13567 | C14567 | C23456 | C23457 | C23467 | C23567 | C24567 | C34567 | C | C | C |
| F2357 | F2367 | F2456 | F2457 | F2467 | F2567 | F3456 | F3457 | F3467 | F3576 | F4567 | F | D12345 | D12346 | D12347 | D12356 | D12357 | D12367 | D12456 | D12457 | D12467 | D12567 | D13456 | D13457 |
| F1347 | F1356 | F1357 | F1367 | F1456 | F1457 | F1467 | F1567 | F2345 | F2346 | F2347 | F2356 | D13467 | D13567 | D14567 | D23456 | D23457 | D23467 | D23567 | D24567 | D34567 | D | D | D |
| S6 II and S6 II copy | | | | | | | | | | | | S7 | | | | | | | | | | | |
| E12345 | E12346 | E12347 | E12356 | E12357 | E12367 | E12456 | E12457 | E12467 | E12567 | E13456 | E13457 | A123456 | A123457 | A123467 | A123567 | A124567 | A134567 | A234567 | CT | CT | CT | CT | CT |
| E13467 | E13567 | E14567 | E23456 | E23457 | E23467 | E23567 | E24567 | E34567 | E | E | E | B123456 | B123457 | B123467 | B123567 | B124567 | B134567 | B234567 | CT | CT | CT | CT | CT |
| F12345 | F12346 | F12347 | F12356 | F12357 | F12367 | F12456 | F12457 | F12467 | F12567 | F13456 | F13457 | C123456 | C123457 | C123467 | C123567 | C124567 | C134567 | C234567 | CT | CT | CT | CT | CT |
| F13467 | F13567 | F14567 | F23456 | F23457 | F23467 | F23567 | F24567 | F34567 | F | F | F | D123456 | D123457 | D123467 | D123567 | D124567 | D134567 | D234567 | CT | CT | CT | CT | CT |
| F12345 | F12346 | F12347 | F12356 | F12357 | F12367 | F12456 | F12457 | F12467 | F12567 | F13456 | F13457 | E123456 | E123457 | E123467 | E123567 | E124567 | E134567 | E234567 | CT | CT | CT | CT | CT |
| F13467 | F13567 | F14567 | F23456 | F23457 | F23467 | F23567 | F24567 | F34567 | F | F | F | F123456 | F123457 | F123467 | F123567 | F124567 | F134567 | F234567 | CT | CT | CT | CT | CT |
| E12345 | E12346 | E12347 | E12356 | E12357 | E12367 | E12456 | E12457 | E12467 | E12567 | E13456 | E13457 | CT | CT | CT | CT | CT | CT | CT | CT | CT | CT | CT | CT |
| E13467 | E13567 | E14567 | E23456 | E23457 | E23467 | E23567 | E24567 | E34567 | E | E | E | CT | CT | CT | CT | CT | CT | CT | CT | CT | CT | CT | CT |

| Table S5 (part 4). 96-well plates layout for screening | | | | | | | | | | | | | | | | | | | | | | | |
| --- | --- | --- | --- | --- | --- | --- | --- | --- | --- | --- | --- | --- | --- | --- | --- | --- | --- | --- | --- | --- | --- | --- | --- |
| S7 copy and S8 / S8 copy | | | | | | | | | | | |  | | | | | | | | | | | |
| A123456 | A123457 | A123467 | A123567 | A124567 | A134567 | A234567 | BK | BK | BK | BK | BK |  |  |  |  |  |  |  |  |  |  |  |  |
| B123456 | B123457 | B123467 | B123567 | B124567 | B134567 | B234567 | BK | BK | BK | BK | BK |  |  |  |  |  |  |  |  |  |  |  |  |
| C123456 | C123457 | C123467 | C123567 | C124567 | C134567 | C234567 | BK | BK | BK | BK | BK |  |  |  |  |  |  |  |  |  |  |  |  |
| D123456 | D123457 | D123467 | D123567 | D124567 | D134567 | D234567 | BK | BK | BK | BK | BK |  |  |  |  |  |  |  |  |  |  |  |  |
| E123456 | E123457 | E123467 | E123567 | E124567 | E134567 | E234567 | BK | BK | BK | BK | BK |  |  |  |  |  |  |  |  |  |  |  |  |
| F123456 | F123457 | F123467 | F123567 | F124567 | F134567 | F234567 | BK | BK | BK | BK | BK |  |  |  |  |  |  |  |  |  |  |  |  |
| A1234567 | B1234567 | C1234567 | D1234567 | E1234567 | F1234567 | BK | BK | BK | BK | BK | BK |  |  |  |  |  |  |  |  |  |  |  |  |
| A1234567 | B1234567 | C1234567 | D1234567 | E1234567 | F1234567 | BK | BK | BK | BK | BK | BK |  |  |  |  |  |  |  |  |  |  |  |  |
| Note: Layout of 96-well plates in the screening experiment. S1-S8 refers to size 1- size 8 consortia,i.e. consortia with 1-8 co-cultured bacteira strains.S1-S8 copy refers to biological replica. A: *Pseudomonas fluorescens*, B: *Rhodococcus erythropolis*, C: *Pseudomonas sp*.,D: *Delftia sp*.,E: *Rhodococcus sp1.*,F: *Rhodococcus sp2.*, 1: *Pseudomonas syringae*, 2: *Agromyces atrinae*, 3: *Cupriavidus metallidurans*, 4: *Ochrobactrum anthropic*, 5: *Plantibacter flavus*, 6: *Plantibacter sp.*, 7: *Rhodococcus sp3.*, CT: axenic algae control (*Coelastrella terrestris*), BK: blank. | | | | | | | | | | | | | | | | | | | | | | | |

8. Non-degrader labels and combination

| Table S6 Non-degrader labels and combination | | | | | | | | | | | | | | | |
| --- | --- | --- | --- | --- | --- | --- | --- | --- | --- | --- | --- | --- | --- | --- | --- |
| **1** | **2** | **3** | **4** | **5** | **6** | **7** | **8** | **9** | **10** | **11** | **12** | **13** | **14** | **15** | **16** |
| N/A | 1 | 2 | 3 | 4 | 5 | 6 | 7 | 1, 2 | 1, 3 | 1, 4 | 1, 5 | 1, 6 | 1, 7 | 2, 3 | 2, 4 |
| **17** | **18** | **19** | **20** | **21** | **22** | **23** | **24** | **25** | **26** | **27** | **28** | **29** | **30** | **31** | **32** |
| 2, 5 | 2, 6 | 2, 7 | 3, 4 | 3, 5 | 3, 6 | 3, 7 | 4, 5 | 4, 6 | 4, 7 | 5, 6 | 5, 7 | 6, 7 | 1, 2, 3 | 1, 2, 4 | 1, 2, 5 |
| **33** | **34** | **35** | **36** | **37** | **38** | **39** | **40** | **41** | **42** | **43** | **44** | **45** | **46** | **47** | **48** |
| 1, 2, 6 | 1, 2, 7 | 1, 3, 4 | 1, 3, 5 | 1, 3, 6 | 1, 3, 7 | 1, 4, 5 | 1, 4, 6 | 1, 4, 7 | 1, 5, 6 | 1, 5, 7 | 1, 6, 7 | 2, 3, 4 | 2, 3, 5 | 2, 3, 6 | 2, 3, 7 |
| **49** | **50** | **51** | **52** | **53** | **54** | **55** | **56** | **57** | **58** | **59** | **60** | **61** | **62** | **63** | **64** |
| 2, 4, 5 | 2, 4, 6 | 2, 4, 7 | 2, 5, 6 | 2, 5, 7 | 2, 6, 7 | 3, 4, 5 | 3, 4, 6 | 3, 4, 7 | 3, 5, 6 | 3, 5, 7 | 3, 6, 7 | 4, 5, 6 | 4, 5, 7 | 4, 6, 7 | 5, 6, 7 |
| **65** | **66** | **67** | **68** | **69** | **70** | **71** | **72** | **73** | **74** | **75** | **76** | **77** | **78** | **79** | **80** |
| 1, 2, 3, 4 | 1, 2, 3, 5 | 1, 2, 3, 6 | 1, 2, 3, 7 | 1, 2, 4, 5 | 1, 2, 4, 6 | 1, 2, 4, 7 | 1, 2, 5, 6 | 1, 2, 5, 7 | 1, 2, 6, 7 | 1, 3, 4, 5 | 1, 3, 4, 6 | 1, 3, 4, 7 | 1, 3, 5, 6 | 1, 3, 5, 7 | 1, 3, 6, 7 |
| **81** | **82** | **83** | **84** | **85** | **86** | **87** | **88** | **89** | **90** | **91** | **92** | **93** | **94** | **95** | **96** |
| 1, 4, 5, 6 | 1, 4, 5, 7 | 1, 4, 6, 7 | 1, 5, 6, 7 | 2, 3, 4, 5 | 2, 3, 4, 6 | 2, 3, 4, 7 | 2, 3, 5, 6 | 2, 3, 5, 7 | 2, 3, 6, 7 | 2, 4, 5, 6 | 2, 4, 5, 7 | 2, 4, 6, 7 | 2, 5, 6, 7 | 3, 4, 5, 6 | 3, 4, 5, 7 |
| **97** | **98** | **99** | **100** | **101** | **102** | **103** | **104** | **105** | **106** | **107** | **108** | **109** | **110** | **111** | **112** |
| 3, 4, 6, 7 | 3, 5, 6, 7 | 4, 5, 6, 7 | 1, 2, 3, 4, 5 | 1, 2, 3, 4, 6 | 1, 2, 3, 4, 7 | 1, 2, 3, 5, 6 | 1, 2, 3, 5, 7 | 1, 2, 3, 6, 7 | 1, 2, 4, 5, 6 | 1, 2, 4, 5, 7 | 1, 2, 4, 6, 7 | 1, 2, 5, 6, 7 | 1, 3, 4, 5, 6 | 1, 3, 4, 5, 7 | 1, 3, 4, 6, 7 |
| **113** | **114** | **115** | **116** | **117** | **118** | **119** | **120** | **121** | **122** | **123** | **124** | **125** | **126** | **127** | **128** |
| 1, 3, 5, 6, 7 | 1, 4, 5, 6, 7 | 2, 3, 4, 5, 6 | 2, 3, 4, 5, 7 | 2, 3, 4, 6, 7 | 2, 3, 5, 6, 7 | 2, 4, 5, 6, 7 | 3, 4, 5, 6, 7 | 1, 2, 3, 4, 5, 6 | 1, 2, 3, 4, 5, 7 | 1, 2, 3, 4, 6, 7 | 1, 2, 3, 5, 6, 7 | 1, 2, 4, 5, 6, 7 | 1, 3, 4, 5, 6, 7 | 2, 3, 4, 5, 6, 7 | 1, 2, 3, 4, 5, 6 |
| Serial labels of combinations of non-degraders are represented by numbers in red bold. Actual compositions/elements of non-degraders are represented by numbers in black non-bold. 1: *Pseudomonas syringae*, 2: *Agromyces atrinae*, 3: *Cupriavidus metallidurans*, 4: *Ochrobactrum anthropic*, 5: *Plantibacter flavus*, 6: *Plantibacter sp.*, 7: *Rhodococcus sp3* | | | | | | | | | | | | | | | |

9.Primers used for qPCR experiments

| Table S7 Primer design/selection for qPCR | | |
| --- | --- | --- |
| Name | Sequence(5’-3’) | Amp. length |
| 1F | CTTCGGGCCTTGCGCTATCA | 449 bp |
| 1R | CTCTAGCTTGCCAGTTTTGG |  |
| 2F | TGAAGGAGAGCTTGCTCTTT | 415 bp |
| 2R | AGCCGGTGCTTTTTCTGCAA |  |
| 3F | AGTAGCTGGTCTGAGAGGAC | 193 bp |
| 3R | CCACGCCAGGTATTAACCAG |  |
| 4F | CGGGGGAAAGATTTATCGGC | 364 bp |
| 4R | AAATCCGAACAACGCTAGCC |  |
| 5F | GAGCTTCCACCGCATGGTGA | 480 bp |
| 5R | CCGCTACACCAGGAATTCCAA |  |
| BF | GTACGGCACCCACCCGGTAA | 400 bp |
| BR | ACTTGGCAGGCAACGTCTTG |  |
| DR | GGCCTTCGGGTTGTAAACTG | 338 bp |
| DF | GTCAGTACAGGTCCAGGGGA |  |
| Primers designed using [NCBI Primer designing tool](https://www.ncbi.nlm.nih.gov/tools/primer-blast/), a custom database of 16s rDNA sequences (or catA gene sequence for *Rhodococcus*) of pre-identified strains was used for primer pair specificity check. *Note: The annealing and extension temperature of primer set 2F-2R was set at 68°C. | | |

Reference

Abraham, M.H., Whiting, G.S., Fuchs, R., et al. (1990) Thermodynamics of solute transfer from water to hexadecane. *Journal of the Chemical Society, Perkin Transactions 2*, 2 (2): 291–300. doi:10.1039/P29900000291.

Alemzadeh, E., Haddad, R., Ahmadi, A.R., et al. (2014) Identification of Chlorophyceae based on 18S rDNA sequences from Persian Gulf. *Iranian Journal of Microbiology*, 6 (6): 437–442. Available at: http://ijm.tums.ac.ir (Accessed: 8 October 2020).

Amézqueta, S., Subirats, X., Fuguet, E., et al. (2020) Octanol-Water Partition Constant. *Liquid-Phase Extraction*, pp. 183–208. doi:10.1016/B978-0-12-816911-7.00006-2.

Cabani, S., Conti, G. and Lepori, L. (1971) Thermodynamic study on aqueous dilute solutions of organic compounds. Part 2.—Cyclic ethers. *Transactions of the Faraday Society*, 67 (0): 1943–1950. doi:10.1039/TF9716701943.

Duchowicz, P.R., Aranda, J.F., Bacelo, D.E., et al. (2020) QSPR study of the Henry’s law constant for heterogeneous compounds. *Chemical Engineering Research and Design*, 154: 115–121. doi:10.1016/J.CHERD.2019.12.009.

Duong, V.T., Li, Y., Nowak, E., et al. (2012) Microalgae isolation and selection for prospective biodiesel production. *Energies*, 5 (6): 1835–1849. doi:10.3390/en5061835.

Harrison, M.A.J., Cape, J.N. and Heal, M.R. (2002) Experimentally determined Henry’s Law coefficients of phenol, 2-methylphenol and 2-nitrophenol in the temperature range 281–302 K. *Atmospheric Environment*, 36 (11): 1843–1851. doi:10.1016/S1352-2310(02)00137-1.

Hiatt, M.H. (2013) Determination of Henry’s law constants using internal standards with benchmark values. *Journal of Chemical and Engineering Data*, 58 (4): 902–908. doi:10.1021/JE3010535/SUPPL_FILE/JE3010535_SI_002.PDF.

Hilal, S.H., Ayyampalayam, S.N. and Carreira, L.A. (2008) Air - liquid partition coefficient for a diverse set of organic compounds: Henry’s law constant in water and hexadecane. *Environmental Science and Technology*, 42 (24): 9231–9236. doi:10.1021/ES8005783/SUPPL_FILE/ES8005783_SI_001.PDF.

Jian, L. (2008) Dynamic Measurements of Henry’s Law Constant of Aromatic Compounds Using Proton Transfer Reaction Mass Spectrometry. *Acta Physico-chimica Sinica*.

Khaw, Y.S., Khong, N.M.H., Shaharuddin, N.A., et al. (2020) A simple 18S rDNA approach for the identification of cultured eukaryotic microalgae with an emphasis on primers. *Journal of Microbiological Methods*, 172: 105890. doi:10.1016/j.mimet.2020.105890.

Kim, Y.H. and Kim, K.H. (2014) Recent advances in thermal desorption-gas chromatography-mass spectrometery method to eliminate the matrix effect between air and water samples: Application to the accurate determination of Henry’s law constant. *Journal of Chromatography A*, 1342: 78–85. doi:10.1016/J.CHROMA.2014.03.040.

Kish, J.D., Leng, C., Kelley, J., et al. (2013) An improved approach for measuring Henry ’ s law coef fi cients of atmospheric organics. *Atmospheric Environment*, 79: 561–565. doi:10.1016/j.atmosenv.2013.07.023.

Kutsuna, S. and Kaneyasu, N. (2021) Henry ’ s law constants and hydration equilibrium constants of n -hexanal and their temperature dependence as determined by the rectangular pulse method. *Chemical Engineering Science*, 239: 116639. doi:10.1016/j.ces.2021.116639.

Lee, S., Mukherjee, S., Brewer, B., et al. (2013) *A Laboratory Experiment To Measure Henry ’ s Law Constants of Volatile Organic Compounds with a Bubble Column and a Gas Chromatography Flame Ionization Detector (GC-FID)*.

Mackay, D., Shiu, W.-Y., Shiu, W.-Y., et al. (2006) Handbook of Physical-Chemical Properties and Environmental Fate for Organic Chemicals. *Handbook of Physical-Chemical Properties and Environmental Fate for Organic Chemicals*. doi:10.1201/9781420044393.

Matsumoto, M., Sugiyama, H., Maeda, Y., et al. (2010) Marine diatom, Navicula sp. strain JPCC DA0580 and marine green alga, Chlorella sp. Strain NKG400014 as potential sources for biodiesel production. *Applied Biochemistry and Biotechnology*, 161 (1–8): 483–490. doi:10.1007/s12010-009-8766-x.

Moldoveanu, S. and David, V. (2015a) *Chapter 6 Solvent Extraction*. Elsevier B.V. Available at: http://www.sciencedirect.com:5070/book/9780444543196/modern-sample-preparation-for-chromatography (Downloaded: 10 October 2023).

Moldoveanu, S. and David, V. (2015b) Phase Transfer in Sample Preparation. *Modern Sample Preparation for Chromatography*, pp. 105–130. doi:10.1016/B978-0-444-54319-6.00005-0.

Rudi, K., Skulberg, O.M., Larsen, F., et al. (1997) Strain characterization and classification of oxyphotobacteria in clone cultures on the basis of 16S rRNA sequences from the variable regions V6, V7, and V8. *Applied and Environmental Microbiology*, 63 (7): 2593–2599. doi:10.1128/aem.63.7.2593-2599.1997.

Schwardt, A., Dahmke, A. and Köber, R. (2021) Henry’s law constants of volatile organic compounds between 0 and 95 °C – Data compilation and complementation in context of urban temperature increases of the subsurface. *Chemosphere*, 272: 129858. doi:10.1016/J.CHEMOSPHERE.2021.129858.

Sieg, K., Starokozhev, E., Schmidt, M.U., et al. (2009) Inverse temperature dependence of Henry’s law coefficients for volatile organic compounds in supercooled water. *Chemosphere*, 77 (1): 8–14. doi:10.1016/J.CHEMOSPHERE.2009.06.028.

Signer, R., Arm, H. and Daeniker, H. (1969) Untersuchungen über das Verhalten organischer Mischphasen 8. Mitteilung. Dampfdrücke, Dichten, thermodynamische Mischungsfunktionen und Brechungsindices der binären Systeme Wasser-Tetrahydrofuran und Wasser-Diäthyläther bei 25°. *Helvetica Chimica Acta*, 52 (8): 2347–2351. doi:10.1002/HLCA.19690520816.

Timmins, M., Thomas-Hall, S.R., Darling, A., et al. (2009) Phylogenetic and molecular analysis of hydrogen-producing green algae. *Journal of Experimental Botany*, 60 (6): 1691–1702. doi:10.1093/jxb/erp052.

TURNER, S., PRYER, K.M., MIAO, V.P.W., et al. (1999) Investigating Deep Phylogenetic Relationships among Cyanobacteria and Plastids by Small Subunit rRNA Sequence Analysis. *The Journal of Eukaryotic Microbiology*, 46 (4): 327–338. doi:10.1111/j.1550-7408.1999.tb04612.x.

Wang, C., Yuan, T., Wood, S., et al. (2017) Uncertain Henry’s law constants compromise equilibrium partitioning calculations of atmospheric oxidation products. *Atmospheric Chemistry and Physics*, 17 (12): 7529–7540. doi:10.5194/ACP-17-7529-2017.
